# Palmitoylation mediates membrane association of hepatitis E virus ORF3 protein and is required for infectious particle secretion

**DOI:** 10.1371/journal.ppat.1007471

**Published:** 2018-12-10

**Authors:** Jérôme Gouttenoire, Angela Pollán, Laurence Abrami, Noémie Oechslin, Johann Mauron, Maxime Matter, Joël Oppliger, Dagmara Szkolnicka, Viet Loan Dao Thi, F. Gisou van der Goot, Darius Moradpour

**Affiliations:** 1 Division of Gastroenterology and Hepatology, Centre Hospitalier Universitaire Vaudois, University of Lausanne, Lausanne, Switzerland; 2 Global Health Institute, Ecole Polytechnique Fédérale de Lausanne, Lausanne, Switzerland; The University of Chicago, UNITED STATES

## Abstract

Hepatitis E virus (HEV) is a positive-strand RNA virus encoding 3 open reading frames (ORF). HEV ORF3 protein is a small, hitherto poorly characterized protein involved in viral particle secretion and possibly other functions. Here, we show that HEV ORF3 protein forms membrane-associated oligomers. Immunoblot analyses of ORF3 protein expressed in cell-free *vs*. cellular systems suggested a posttranslational modification. Further analyses revealed that HEV ORF3 protein is palmitoylated at cysteine residues in its N-terminal region, as corroborated by ^3^H-palmitate labeling, the investigation of cysteine-to-alanine substitution mutants and treatment with the palmitoylation inhibitor 2-bromopalmitate (2-BP). Abrogation of palmitoylation by site-directed mutagenesis or 2-BP treatment altered the subcellular localization of ORF3 protein, reduced the stability of the protein and strongly impaired the secretion of infectious particles. Moreover, selective membrane permeabilization coupled with immunofluorescence microscopy revealed that HEV ORF3 protein is entirely exposed to the cytosolic side of the membrane, allowing to propose a model for its membrane topology and interactions required in the viral life cycle. In conclusion, palmitoylation determines the subcellular localization, membrane topology and function of HEV ORF3 protein in the HEV life cycle.

## Author summary

Hepatitis E virus (HEV) infection is believed to be the most common cause of acute hepatitis and jaundice in the world. HEV is a positive-strand RNA virus found as a non-enveloped virion in bile and feces or as a quasi-enveloped virion in blood and in cell culture. The HEV ORF3 protein is involved in viral particle secretion likely through the exosomal pathway. Here, we provide evidence for palmitoylation of ORF3 protein at its N-terminal cysteine-rich domain. Palmitoylation of ORF3 protein determines its subcellular localization and function in particle secretion. In addition, our data indicate a membrane topology where HEV ORF3 protein is entirely exposed to the cytosol, providing important insight into its interactions in the viral life cycle.

## Introduction

Hepatitis E virus (HEV) infection is believed to be the most common cause of acute hepatitis and jaundice in the world [1, 2]. It is a positive-strand RNA virus classified in the *Hepeviridae* family [3, 4]. Most human pathogenic strains can be assigned to genotypes 1–4 within the species *Orthohepevirus A* [5]. HEV genotypes 1 and 2 are transmitted by the fecal-oral route and can cause large, primarily waterborne outbreaks in low-income countries with poor sanitation, infecting about 20 million people and claiming 70,000 lives every year [6]. On the other hand, infection with HEV genotypes 3 and 4 has been recognized as a porcine zoonosis in high-income countries, with much higher than anticipated seroprevalence rates, reaching 86.4% in the South of France [7]. HEV genotype 3 (and, to a lesser extent, genotypes 4 as well as 7) can persist in immunocompromised patients and cause chronic hepatitis with potential rapid progression to cirrhosis [8, 9]. In addition, HEV genotype 3 is a cause of diverse neurological manifestations, especially neuralgic amyotrophy (also known as Parsonage-Turner syndrome) [10, 11]. Hence, HEV represents a growing global health concern [12–14].

HEV is a nonenveloped virus of 27–34 nm in diameter. The 7.2-kb RNA genome encodes three open reading frames (ORF) which are translated into (i) the ORF1 polyprotein, representing the viral replicase, (ii) the ORF2 protein, corresponding to the viral capsid, and (iii) the ORF3 protein, a small, hitherto poorly characterized protein [3]. Although HEV is found as a nonenveloped virus in bile and feces, it circulates in the bloodstream, similar to hepatitis A virus, as a 'quasi-enveloped' particle wrapped in cellular membranes likely derived from exosomes [15–17].

HEV ORF3 protein plays an essential role in virion secretion [18, 19] but not in RNA replication or virion assembly [20]. More specifically, ORF3 protein supports HEV egress using the exosomal pathway [16, 21] and has been shown to be associated with the quasi-enveloped viral particle [22]. Interestingly, a recent study reported that HEV ORF3 protein possesses ion channel activity required for the release of infectious virus [23].

ORF3 encodes, depending on the genotype, a 112-, 113- or 114-amino-acid (aa) protein with a predicted molecular weight (MW) of 12–13 kDa. It has been reported to be phosphorylated at Ser 71 in genotype 1 (corresponding to Ser 70 in genotype 3) by the cellular mitogen-activated protein kinase [24]. A yeast two-hybrid study suggested oligomerization of ORF3 protein through a proline-rich C-terminal region [25], and homotypic interaction of ORF3 protein was recently confirmed in mammalian cells [23]. Moreover, phosphorylated ORF3 protein was reported to interact with the ORF2 (capsid) protein [26].

Early reports indicated that ORF3 protein associates, *via* an N-terminal hydrophobic domain, with the cytoskeleton and, more specifically, with microtubules [24, 27]. In addition, the protein has also been observed at early and recycling endosomes [28] as well as multivesicular bodies (MVBs) [16]. Notably, a conserved PSAP motif may be necessary for its interaction with tumor susceptibility gene 101 (Tsg101), a component of the endosomal sorting complexes required for transport (ESCRT) pathway [19, 21, 29].

In the present study, we describe the oligomerization as well as the association of HEV ORF3 protein with intracellular and plasma membranes. We identified a determinant for oligomerization at the N-terminal end of ORF3 protein. Furthermore, we demonstrate palmitoylation of ORF3 protein on cysteine residues in its N-terminal segment. Alanine substitution of the cysteine residues or inhibition of palmitoylation by 2-bromopalmitate (2-BP) lowered the MW of the viral protein and altered ORF3 protein subcellular localization. Importantly, serine substitution of 5 cysteine residues in a full-length HEV clone strongly impaired the secretion of infectious particles. Finally, selective membrane permeabilization coupled with immunofluorescence analyses allowed to determine the membrane topology of HEV ORF3 protein.

## Results

### HEV ORF3 protein oligomerizes through N-terminal determinants

**Fig 1:**
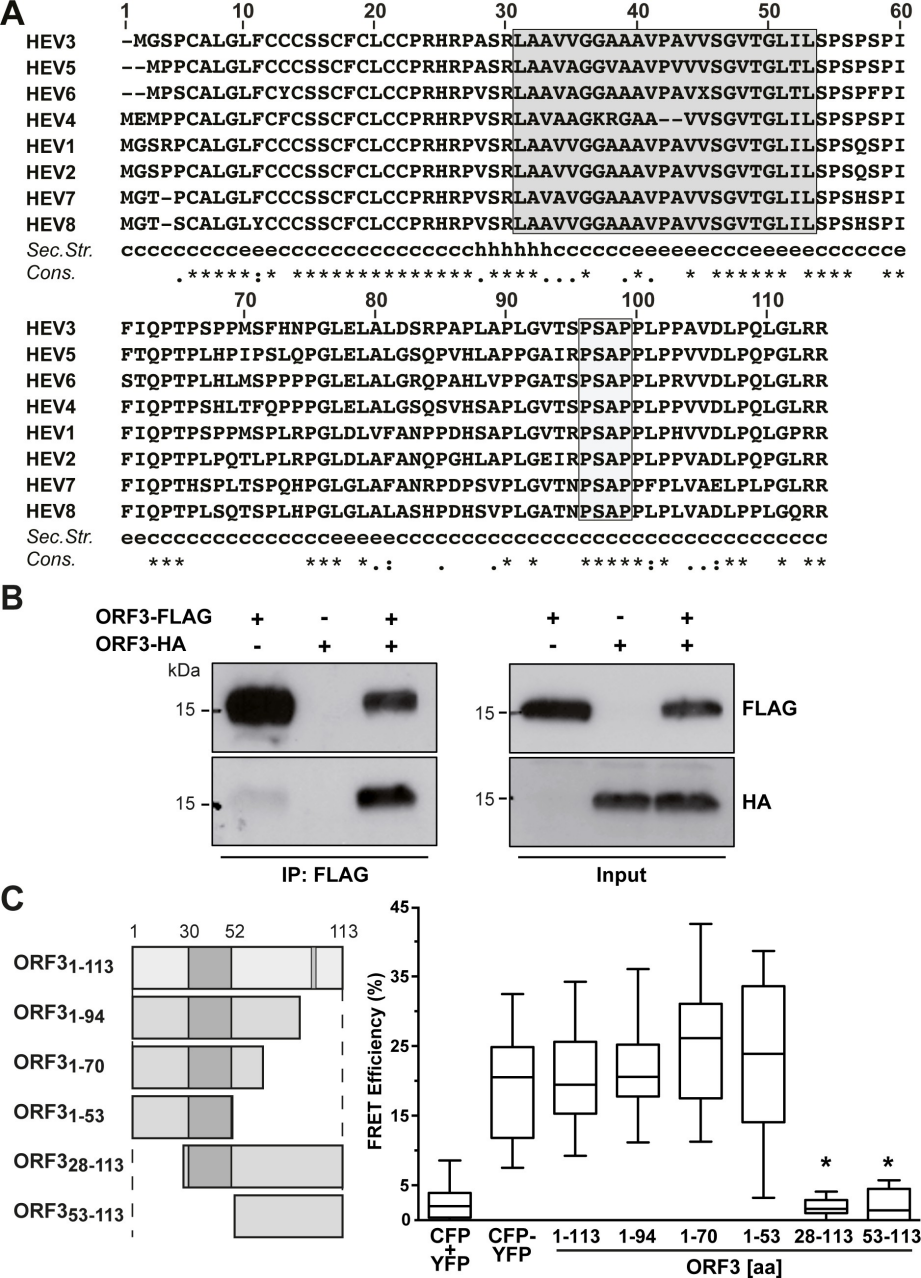
HEV ORF3 protein oligomerizes in mammalian cells. (**A**) Sequence analysis of ORF3 protein. Amino acid sequences of ORF3 from HEV genotypes 1–8 (GenBank accession numbers AB740232, AF444002, M74506, AJ272108, AB573435, AB856243, KJ496144 and KX387866) were aligned using ClustalW [52]. Segment aa 30–53 predicted as transmembrane passage by TMPred is boxed in grey [53]. A consensus secondary structure was predicted using algorithms MLRC, DSC and PHD (available at https://npsa-prabi.ibcp.fr) and is shown below the alignment (c, random coil; h, α-helix; e, extended strand;?, discrepant prediction). The degree of aa physicochemical conservation at each position is shown on the bottom line and can be inferred with the similarity index according to ClustalW convention (asterisk, invariant; colon, highly similar; dot, similar) [52]. (**B**) ORF3 protein oligomerization was analyzed by FLAG immunoprecipitation. Lysates (Input) of U-2 OS cells transfected with pCMVORF3-HA and/or pCMVORF3-FLAG as well as immunoprecipitates (IP: FLAG) were subjected to immunoblot with either specific anti-FLAG or anti-HA antibodies. The presence of a strong signal for ORF3-HA after pull-down of ORF3-FLAG indicates oligomerization of ORF3 protein in cells. (**C**) FRET analyses reveal oligomerization of HEV ORF3. CFP (cyan fluorescent protein) or YFP (yellow fluorescent protein) fused to the C-termini of HEV ORF3 segments aa 1–113, 1–94, 1–70, 1–53, 28–113 or 53–113 were co-expressed in U-2 OS cells. FRET analyses were performed by the acceptor photobleaching method as described in the Materials and Methods section. The CFP-YFP fusion protein and cotransfection of unfused CFP and YFP served as positive and negative controls, respectively. Box-and-whisker plots represent the median FRET efficiency (FRETeff) values of 20 measurements (middle line), the values from the lower to the upper quartile (central box), and the minimum and maximum values (vertical line). The significance of the observed differences was assessed as described in Materials and Methods (*, P<0.0001).

The N-terminal region of ORF3 protein has previously been reported to harbor two hydrophobic segments of which the second has been predicted as a transmembrane segment [23]. Sequence analysis of HEV ORF3 from all 8 genotypes revealed that the protein sequence is highly conserved especially in the N-terminal part (aa 1–28) (Fig 1A). Of note, the predicted transmembrane segment, boxed in grey in Fig 1A (segment aa 30–53 for gt 3), shows more variability in primary sequence, especially in gt 4 as compared to the other genotypes, and, intriguingly, is not predicted to form an α-helix.

HEV ORF3 protein has been reported to oligomerize in a yeast two-hybrid study as well as in mammalian cells [23, 25]. However, the determinants for oligomerization need to be further delineated. As shown in Fig 1B, we confirmed oligomerization of ORF3 protein by coimmunoprecipitation of HA- and FLAG-tagged ORF3 constructs in transiently transfected U-2 OS cells. On this basis, we developed a fluorescence resonance energy transfer (FRET) assay to further explore oligomerization of ORF3. A CFP-YFP fusion protein and cotransfection of CFP and YFP served as positive and negative controls, respectively. As shown in Fig 1C, coexpression of CFP- and YFP-tagged full-length ORF3 protein (ORF3_1-113_) constructs yielded a significant FRET signal as compared to the negative and positive controls. Moreover, analyses of C- and N-terminal deletion constructs revealed that the N-terminal 28 aa are essential for the oligomerization of HEV ORF3 protein.

### HEV ORF3 protein associates with membranes

**Fig 2:**
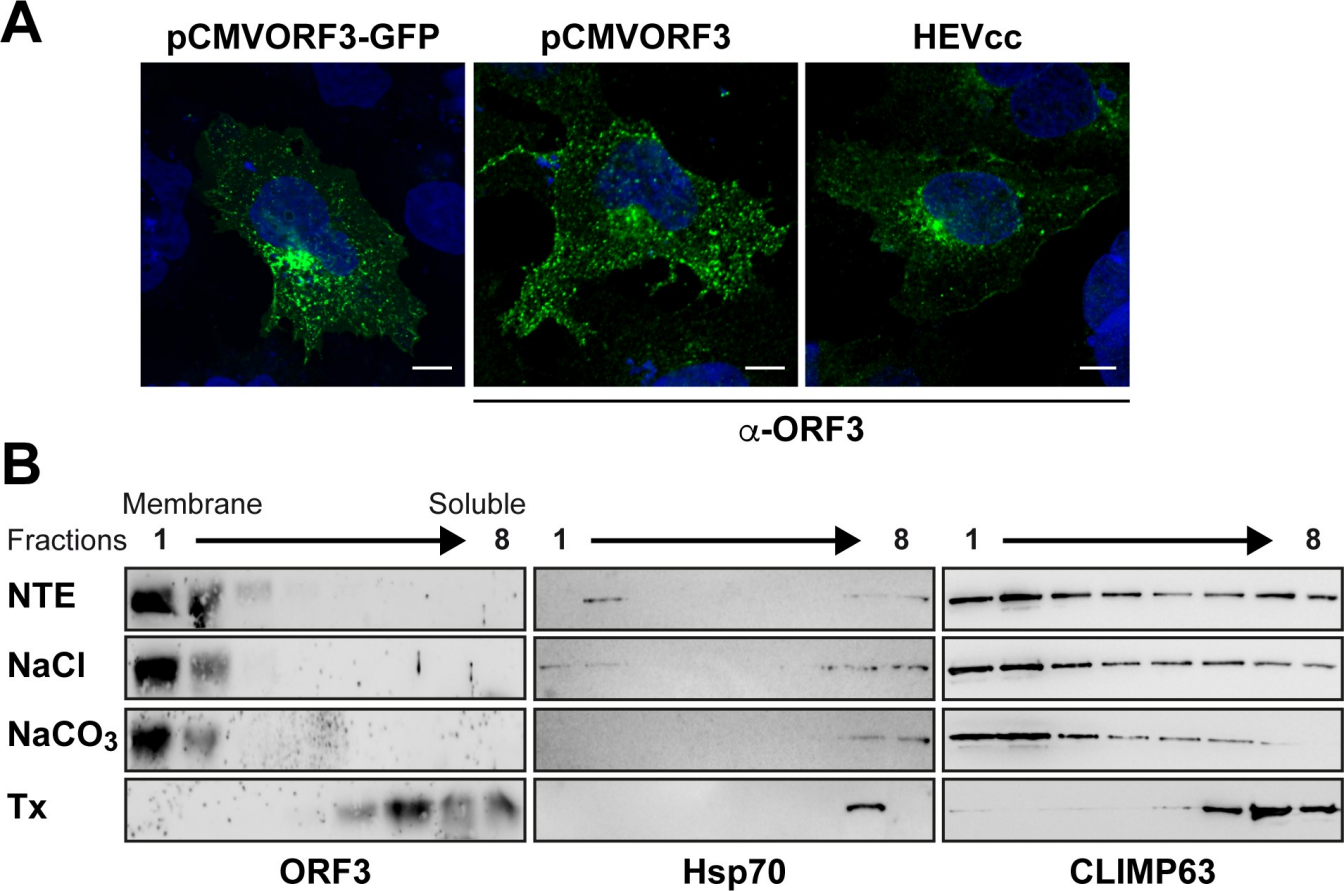
HEV ORF3 protein associates with membranes. (**A**) HEV ORF3 protein is expressed in cell membrane compartments. S10-3 cells were transfected with pCMVORF3-GFP, pCMVORF3, or with full-length p6 HEV RNA (HEVcc). Cells were fixed 48 h or 6 d (HEVcc) post-transfection and analyzed by fluorescence microscopy either by direct visualization of GFP or after immunofluorescence staining of HEV ORF3 protein using anti-ORF3 rabbit pAb (α-ORF3). Scale bars indicate 10 μm. (**B**) Membrane flotation confirms the membrane association of HEV ORF3 protein. S10-3 cells transiently transfected with pCMVORF3 were lysed in a hypotonic buffer and the membrane fraction was obtained by ultracentrifugation as described in the Materials and Methods section. Membrane pellets were resuspended in physiological NTE buffer, 1 M NaCl, 100 mM sodium carbonate (pH 11.5), or 1% Triton X-100 (Tx) and incubated for 20 min at 4°C. Subsequently, membrane flotation analyses were performed as described in the Materials and Methods section. Eight fractions were collected from the top and analyzed by immunoblot using pAb against HEV ORF3. Detection of Hsp70 served as a control for a peripherally membrane-associated protein and CLIMP63 as a control for an integral membrane protein.

ORF3 expressed as a GFP fusion protein or as an untagged protein either individually or in cells replicating full-length infectious HEV is consistently observed at intracellular membrane compartments, likely corresponding to the previously described localization in endosomes and in MVBs [16, 28], but also at the plasma membrane (Fig 2A). Membrane extraction with 1 M NaCl, sodium carbonate pH 11.5, or 1% Triton X-100, followed by flotation assay confirmed that ORF3 protein is tightly associated with cellular membranes (Fig 2B), as indicated by a similar behaviour of ORF3 protein than as compared to the integral endoplasmic reticulum membrane protein CLIMP63.

### The N-terminal region of HEV ORF3 protein determines its membrane association and subcellular localization

**Fig 3:**
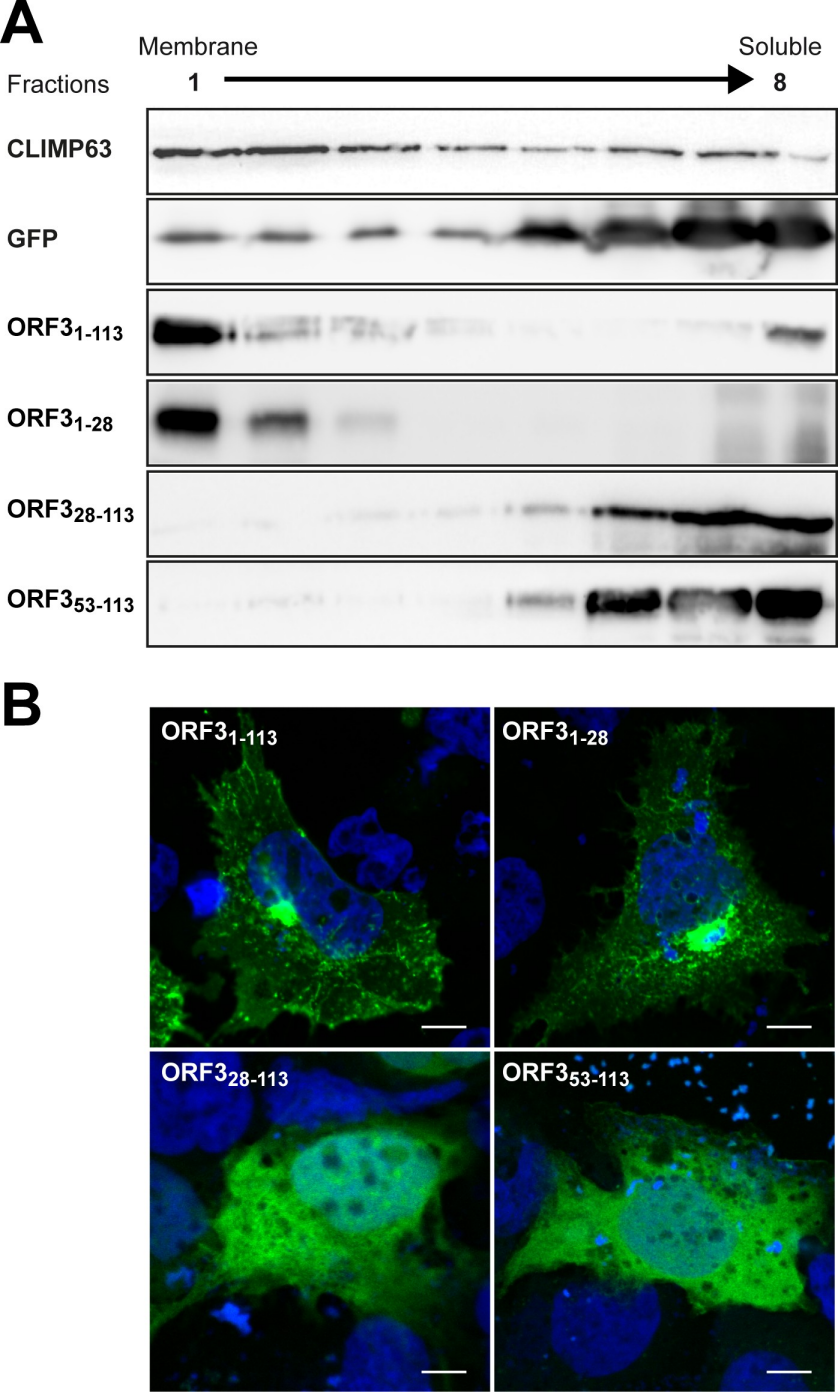
The N-terminal region of HEV ORF3 protein determines its membrane-association and plasma membrane localization. (**A**) Membrane flotation analyses of ORF3 and deletion constructs. S10-3 cells were transiently transfected with pCMVORF3-GFP, pCMVORF3_1-53_-GFP, pCMVORF3_1-28_-GFP, pCMVORF3_28-113_-GFP, pCMVORF3_53-113_-GFP or pCMV-GFP as indicated. Cell lysates were subjected to membrane flotation assay followed by immunoblot analysis using mAb JL8 against GFP, as described in the Material and Methods section. Endogenous CLIMP63 was used as positive control for an integral membrane protein while GFP served as control for a soluble protein. (**B**) Subcellular localization of ORF3-GFP fusion constructs. S10-3 cells were transiently transfected with pCMVORF3-GFP, pCMVORF3_1-53_-GFP, pCMVORF3_1-28_-GFP or pCMVORF3_1-53_-GFP as indicated. Nucleus was stained with DAPI. Scale bars indicate 10 μm. Slides were analyzed by confocal laser scanning microscopy.

To determine the domain responsible for membrane association of HEV ORF3 protein, we analyzed GFP-fused deletion constructs by membrane flotation assay. The endoplasmic reticulum-resident integral membrane protein CLIMP63 and GFP alone were used as reference for membrane-associated and soluble proteins, respectively (Fig 3A). First, we confirmed that ORF3_1-113_-GFP is associated with membranes, similarly to ORF3 protein alone (cf. Fig 2B). As shown in Fig 3A, the N-terminal 28 aa (ORF3_1-28_-GFP) were sufficient to mediate membrane association of ORF3 protein. Deletion of the N-terminal 27 aa (ORF3_28-113_-GFP) or 52 aa (ORF3_53-113_-GFP) completely abolished membrane association. Hence, the N-terminal 28 aa are required for proper membrane association of HEV ORF3 protein.

At the subcellular level, fluorescence microscopy showed that ORF3_1-28_-GFP had a very similar localization than ORF3_1-113_-GFP (Fig 3B) while ORF3_28-113_-GFP and ORF3_53-113_-GFP were displayed predominantly a diffuse pattern. These results show that in addition to the oligomerization of ORF3 protein, the N-terminal region and more particularly aa 1–28 determine the membrane association and subcellular localization of ORF3 protein.

### HEV ORF3 protein is posttranslationally modified on cysteine residues of its N-terminal segment

**Fig 4:**
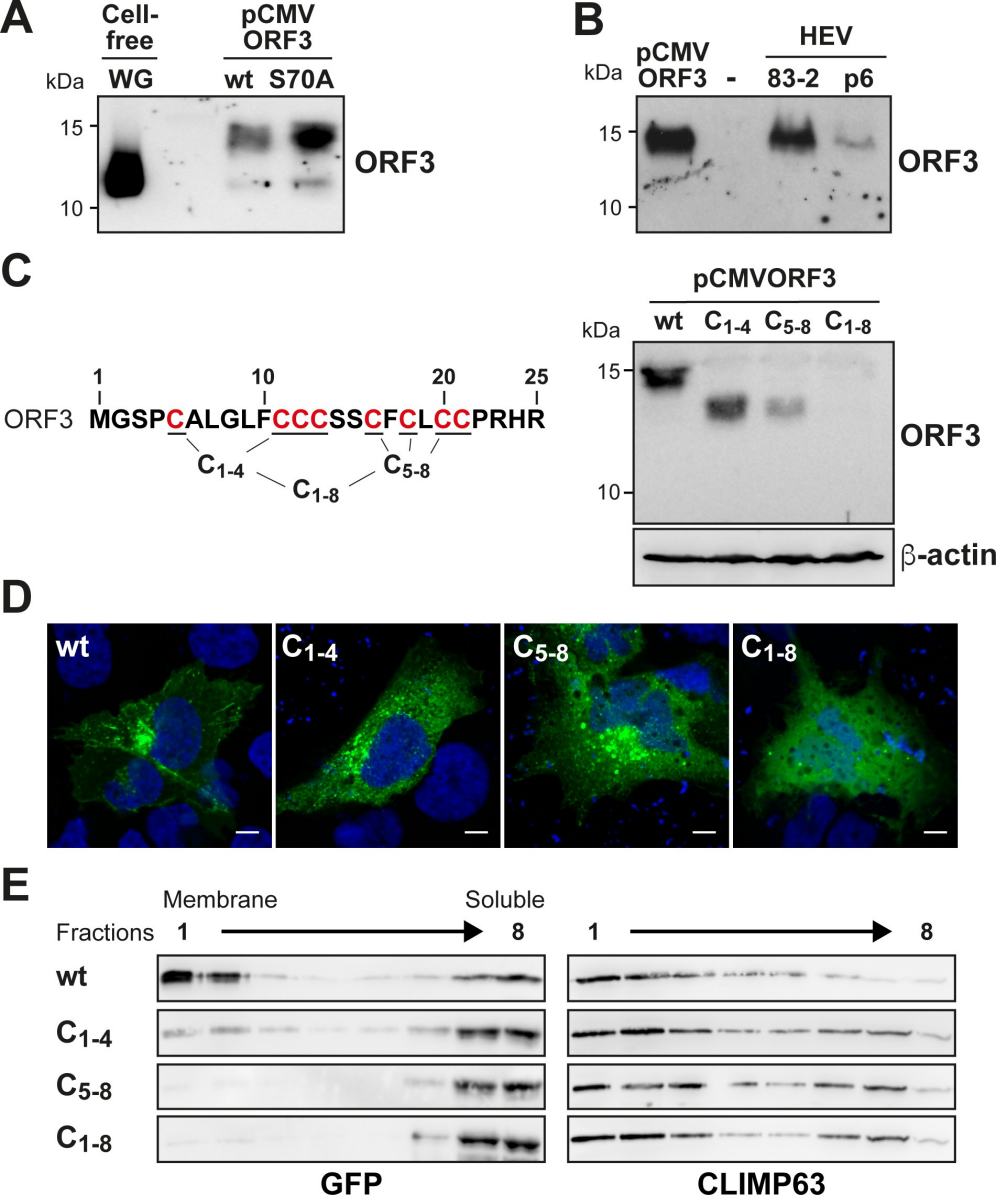
HEV ORF3 protein is posttranslationally modified at the N-terminal cysteine-rich segment. (**A**) S10-3 cells were transfected with pCMVORF3 or pCMVORF3_S70A_. Protein lysates obtained 48 h post-transfection as well as ORF3 protein expressed using wheat germ extract (WG) were separated by 17% SDS-PAGE and subjected to immunoblot with anti-ORF3 pAb. (**B**) Protein lysates from pCMVORF3-transfected cells, naïve Hep293TT cells (-) and cells replicating the full-length p6 or 83–2 HEV clone were prepared 6 days post-electroporation. The samples were separated by 17% SDS-PAGE and subjected to immunoblot with anti-ORF3 pAb. (**C**) Alanine substitution of cysteine residues of HEV ORF3 protein limits its posttranslational modification. S10-3 cells transfected with pCMVORF3, pCMVORF3_C1-4_, pCMVORF3_C45-8_ or pCMVORF3_C1-8_ were harvested 48 h post-transfection and protein lysates were analyzed by immunoblot with anti-ORF3 pAb. (**D**) S10-3 cells transfected with pCMVORF3-GFP, pCMVORF3_C1-4_-GFP, pCMVORF3_C45-8_-GFP or pCMVORF3_C1-8_-GFP were analyzed by fluorescence microscopy 48 h post-transfection. A representative image is shown for each construct. Scale bars indicate 10 μm. (**E**) Membrane flotation analyses of wild-type and mutant ORF3-GFP constructs. S10-3 cells were transiently transfected with pCMVORF3-GFP, pCMVORF3_C1-4_-GFP, pCMVORF3_C45-8_-GFP or pCMVORF3_C1-8_-GFP, as indicated. Cell lysates were subjected to membrane flotation assay followed by immunoblot analysis using mAb JL8 against GFP. Endogenous CLIMP63 was used as positive control for an integral membrane protein.

The 113-aa ORF3 protein of HEV genotype 3 has a theoretical molecular weight (MW) of 11.3 kDa. However, separation of lysates from pCMVORF3-transfected mammalian cells by 17% SDS-PAGE followed by immunoblot analysis revealed an apparent MW of about 15 kDa (Fig 4A). Interestingly, ORF3 protein produced in wheat germ-based cell-free expression system migrated at the expected theoritical MW of about 11–12 kDa (Fig 4A). This observation suggests that ORF3 may undergo posttranslational modification when expressed in cells. As it has been postulated that ORF3 may be phosphorylated, we prepared an alanine substitution mutant (S70A) to determine whether phosphorylation reported at this particular serine residue was involved in the observed MW change. However, as the pattern obtained for this mutant was similar to that of wild-type (wt) ORF3 protein, phosphorylation on Ser 70 does not explain the higher apparent MW observed in our experimental settings (Fig 4A).

Bands other than the one at 15 kDa were observed at lower MWs, including one with a similar migration as compared to cell-free expressed ORF3 protein, i.e. likely the non-modified form. Hence, we validated our observations in lysates from cells replicating infectious HEV from two different molecular clones, i.e 83–2 and p6. As shown in Fig 4B, a single band for ORF3 protein similar to the higher MW signal obtained with the single ORF3 expression was observed under these conditions. Altogether these data show that HEV ORF3 protein has a higher apparent MW than the theoretical, which is likely the consequence of a posttranslational modification.

**Fig 5:**
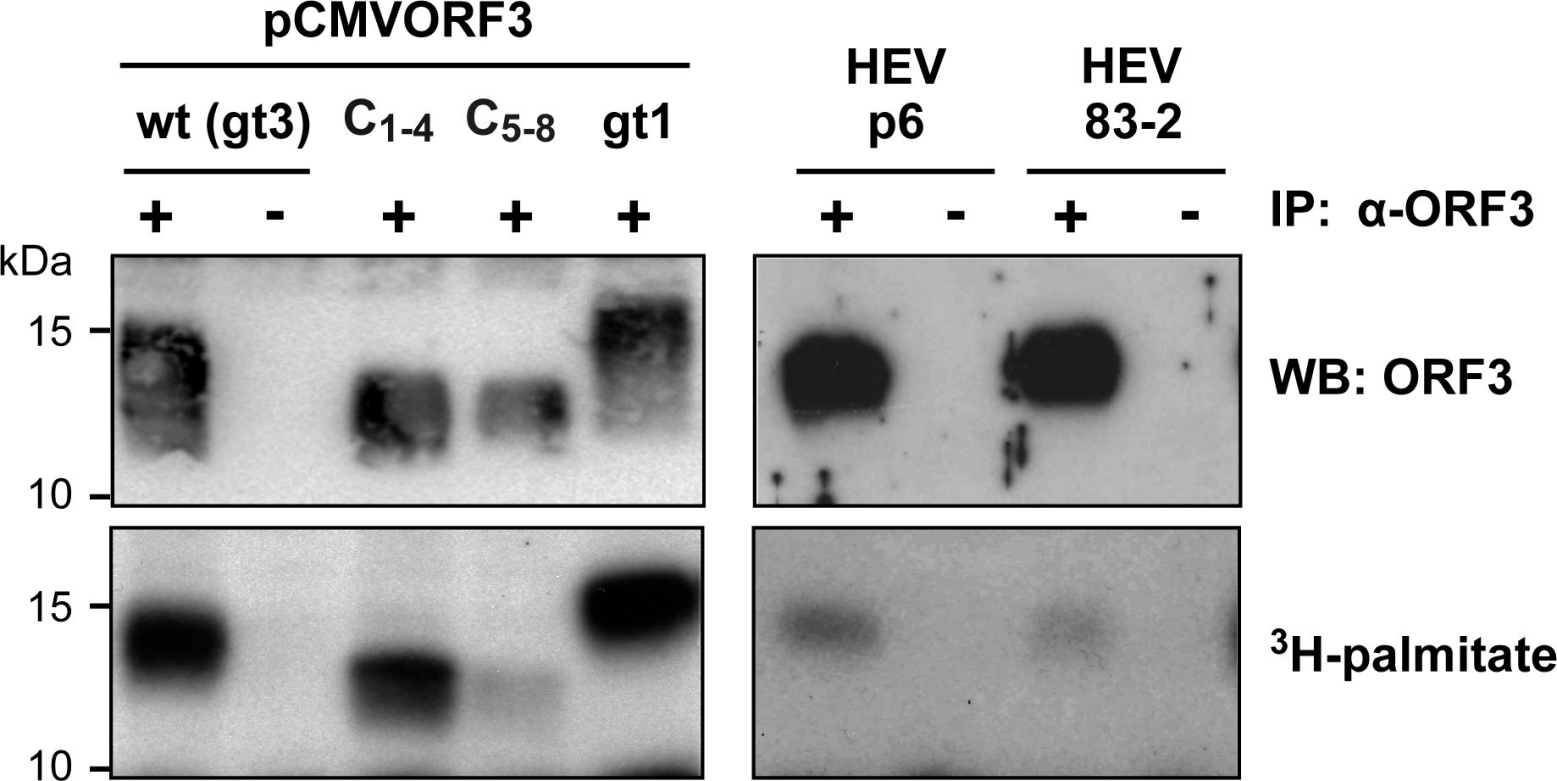
HEV ORF3 protein is palmitoylated. Protein lysates from S10-3 cells transfected with pCMVORF3, pCMVORF3_C1-4_, pCMVORF3_C45-8_ or pCMVORF3_gt1 and from Hep293TT cells replicating the full-length p6 or 83–2 HEV clone were prepared 1 or 6 days post-transfection, respectively, and subjected to immunoprecipitation with either anti-ORF3 pAb (+) or non-relevant rabbit serum (-). After immunoprecipitation, the elution samples were separated by 17% SDS-PAGE and subjected to either immunoblot with anti-ORF3 pAb followed by chemiluminescence revelation or autoradiography (40 d of exposure).

Because an ORF3 protein construct deleted from the first 18 aa displayed a MW which is close to the expected theoretical MW (S1B Fig), we focused our attention on the N-terminal region of ORF3. Interestingly, the first N-terminal 21 aa of the ORF3 protein comprise 8 cysteine residues at conserved positions in all HEV genotypes with the exception of HEV genotypes 4 and 6 which have only 7 cysteines (Fig 1A). Hence, we next substituted the cysteine residues by alanines and analyzed the resulting constructs by immunoblot. Single alanine substitutions resulted in a slight MW shift for all cysteine residues (S1E Fig). Mutants with grouped alanine substitutions, i.e. C_1-4_, C_5-8_ and C_1-8_, showed a more pronounced effect on the MW of ORF3 protein (Fig 4C). Indeed, mutants C_1-4_ and C_5-8_ showed a lower apparent MW and also a reduced protein amount for the latter. Strikingly, alanine substitution of all 8 cysteine residues (mutant C_1-8_) destabilized the protein which could no longer be detected by immunoblot (Fig 4C). Stability of these mutant proteins could be increased by fusion with GFP, as shown in S1F Fig. As shown in Fig 5D, the subcellular localization of ORF3 protein changed upon alanine substitution of the cysteines, i.e. mutants C_1-4_, C_5-8_ or C_1-8_ (Fig 4D), and showed a pattern similar to that of ORF3_28-113_-GFP when all cysteines are mutated (Fig 3B). Furthermore, this observation is correlated with a loss of membrane association of the mutants as assessed by membrane flotation (Fig 4E).

Together, these findings indicate that the cysteine residues of ORF3 protein are likely engaged in a posttranslational modification which plays an important role for the subcellular localization, membrane association and stability of the protein.

### HEV ORF3 protein is palmitoylated

Among common posttranslational modifications involving cysteine residues, palmitoylation results in the covalent link of a palmitate, i.e. a C16 acyl chain. A consequence of the addition of palmitate to cysteine residues is the association with cellular membranes. This modification is mediated by host palmitoyltransferases. To assess whether ORF3 protein is palmitoylated, cells replicating the infectious HEV clones p6 and 83–2 as well as cells transfected with wt, C_1-4_ and C_5-8_ ORF3 expression constructs were incubated for 3 h with ^3^H-palmitate before harvesting and immunoprecipitation with anti-ORF3 pAb. Immunoblot analysis revealed that comparable amounts of ORF3 protein were specifically immunoprecipitated in the different conditions (Fig 5, top panel). Furthermore, the MW observed for the C_1-4_ and C_5-8_ mutants was lower as compared to the wt ORF3 protein, as expected and previously observed (Fig 4C). ^3^H-palmitate incorporation was then assessed by separation of protein lysates under the same conditions, followed by autoradiography. As shown in Fig 5, ORF3 protein incorporated palmitate, as revealed by a strong signal for cells transfected with pCMVORF3 from both gt1 and gt3. Importantly, palmitate incorporation into ORF3 protein is also observed in lysates from cells harboring the infectious HEV clones, albeit at a much lower intensity, reflecting a low activity of translation of ORF3 during the limited 3-h period of ^3^H-palmitate incubation (Fig 5, lower panel). Of note, a radioactive signal is also recorded for the for C_1-4_ and C_5-8_ mutants, indicating that, although harboring 4 alanine substitutions each, they still incorporate palmitate, likely at the 4 remaining cysteine residues. Altogether, these observations demonstrate that ORF3 protein is palmitoylated when expressed as a single protein as well as, more importantly, in the context of the replication of full-length infectious HEV.

### Palmitoylation of ORF3 protein determines its subcellular localization

**Fig 6:**
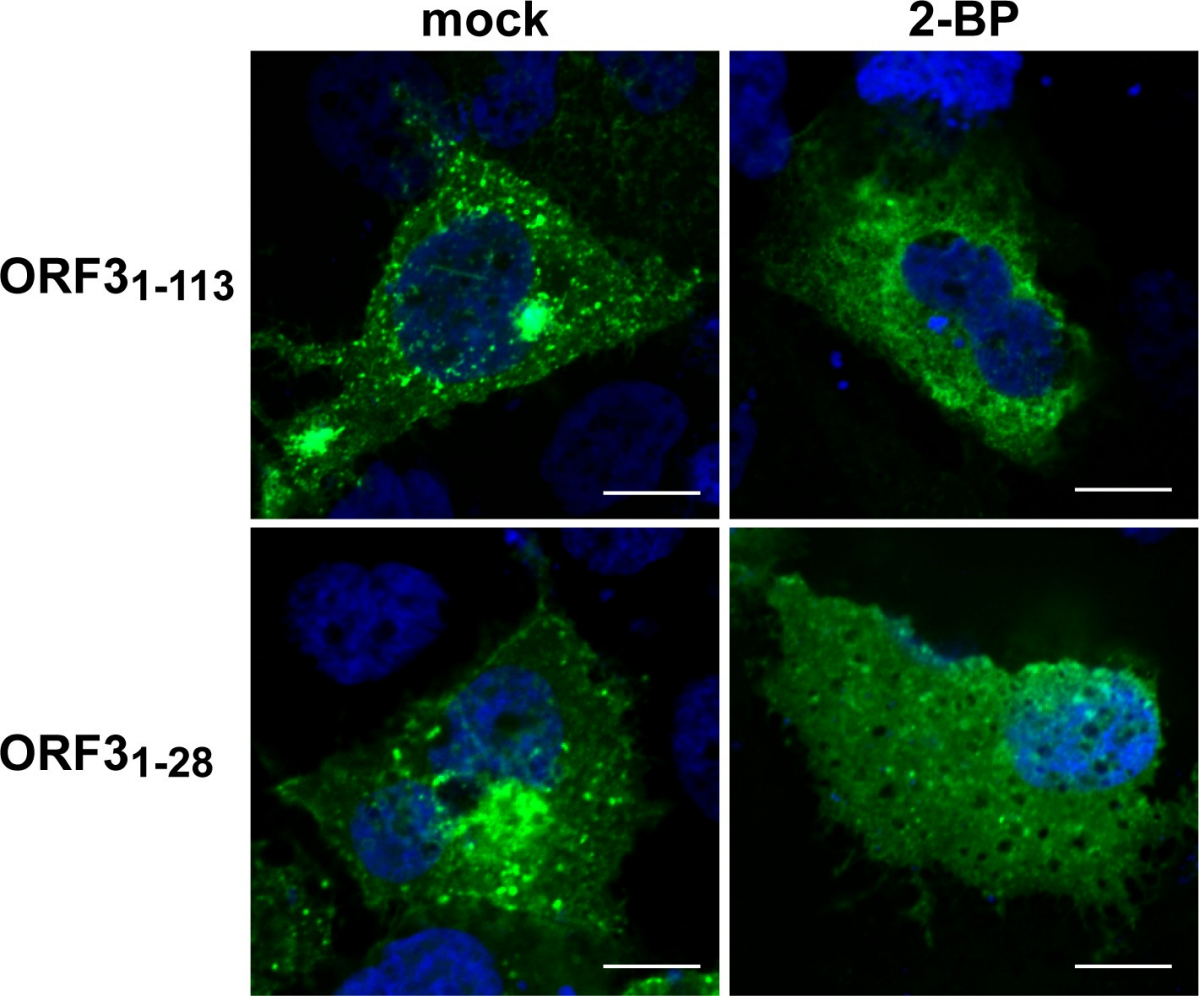
Palmitoylation inhibition induces changes in the subcellular localization of HEV ORF3 protein. S10-3 cells were transfected with either pCMVORF3-GFP (ORF3_1-113_) or pCMVORF3_1-28_-GFP (ORF3_1-28_). One d post-transfection, cells were treated for 24 h with 25 μM 2-bromopalmitate (2-BP) and analyzed by confocal laser scanning microscopy after staining of the nuclei by DAPI. Scale bars represent 10 μm.

We next treated ORF3-expressing cells with increasing concentrations of the palmitoylation inhibitor 2-BP and analyzed the protein lysates for ORF3 protein by immunoblot (S3 Fig). Protein analysis of cells transfected with the pCMVORF3 construct and cultured with increasing concentrations of 2-BP (0.5, 5, 25 and 50 μM) revealed a dose-dependent accumulation of the lower MW signal corresponding to the non-modified ORF3 protein while the higher MW signal was decreasing in parallel (S3 Fig). To determine the role of ORF3 palmitoylation in its subcellular localization, S10-3 cells transfected with GFP fusion constructs were treated with 25 μM 2-BP or DMSO as control for 24 h. As shown in Fig 6, treatment with the palmitoylation inhibitor induced a relocalization of ORF3-GFP within the cytoplasm as a diffuse signal (Fig 6). Furthermore, cells expressing the minimal membrane-associated construct ORF3_1-28_-GFP showed a similar diffuse pattern of the GFP signal within the cytoplasm (Fig 6).

### Palmitoylation of ORF3 protein is essential for secretion of infectious HEV

**Fig 7:**
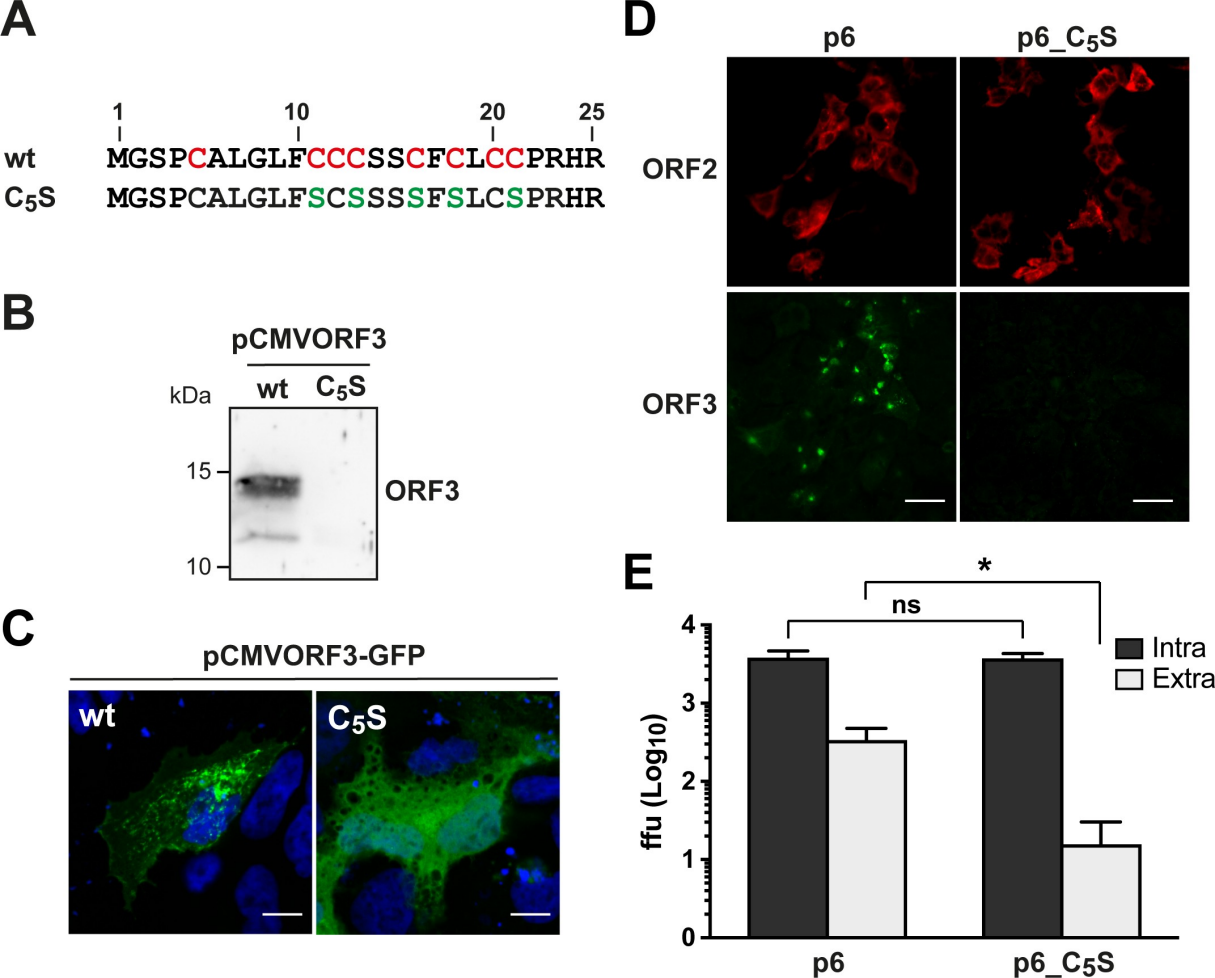
N-terminal cysteine residues of HEV ORF3 protein are essential for infectious particle secretion. (**A**) Sequence alignment of the N-terminal 25 aa of wild-type (wt) and cysteine to serine mutant (C_5_S) HEV ORF3 protein. The conserved cysteine residues in the wt sequence are highlighted in red and the positions bearing a serine substitution in the C_5_S mutant sequence are indicated in green. (**B**) S10-3 cells were transfected with pCMVORF3 or pCMVORF3_C5S_ and cell lysates were prepared 48 h post-transfection, followed by SDS-PAGE and immunoblot analysis using anti-ORF3 pAb. (**C**) S10-3 cells were transiently transfected with pCMVORF3-GFP or pCMVORF3_C5S_-GFP and analyzed by confocal laser scanning microscopy. Nuclei were stained with DAPI. Scale bars indicate 20 μm. (**D**) Indirect immunofluorescence detection of HEV ORF2 and ORF3 proteins was carried out 5 d post-transfection with HEV ORF2 pAb (red) and anti-ORF3 mAb MRB198 (green). Scale bars indicate 50 μm. (**E**) Five d after transfection of p6 or p6_C_5_S HEV RNA, culture supernatants were harvested and cell lysates were prepared by three freeze-and-thaw cycles followed by clarification by centrifugation at 2,000 g for 15 min. Intracellular (Intra) and extracellular (Extra) infectivities were determined by foci forming assay on HepG2/C3A cells using the cell lysates and the culture supernatants, respectively, as inoculum. Immunofluorescence detection of the capsid protein was performed with rabbit antiserum against HEV ORF2. The asterisk (*) indicates statistically significant results with p<0.0001 while “ns” indicates nonsignificant results. Each bar represents infectivity obtained from 10 independent RNA transfections. ffu: focus forming unit.

To investigate the role of palmitoylation in the function of HEV ORF3 protein, 5 cysteine residues were replaced by serine in the full-length HEV p6 infectious clone, yielding mutant p6_C_5_S (Fig 7A). Because ORF2 and ORF3 are overlapping, the cysteine-rich coding region tolerates minimal sequence changes. Therefore, aa at positions 11, 13, 16, 18 and 21 were substituted by a serine residue. Similarly to what was observed for the C_1-8_ mutant, where the 8 cysteine residues have been substituted in a heterologous expression setting, the ORF3_C_5_S protein was not detectable by immunoblot (Fig 7B) and the corresponding GFP fusion construct showed a diffuse subcellular distribution (Fig 7C). Transfection of S10-3 cells with either p6 or p6_C_5_S RNA resulted in the immunofluorescence detection of similar amounts of ORF2 protein while ORF3 protein was detectable only for the wt (Fig 7D). This was also confirmed by immunoblot analysis (S5B Fig). Functional consequences were assessed by the determination of the infectivity in the intracellular and extracellular compartments 5 d post-transfection for the p6 wt and p6_C_5_S constructs. While a similar infectivity was observed intracellularly for both constructs, infectious virus secretion was strongly impaired in the p6_C_5_S mutant (Fig 7E). These results indicate that the cysteine residues of HEV ORF3 protein engaged in palmitoylation are essential for the secretion of infectious particles.

### HEV ORF3 protein is cytosolically oriented

**Fig 8:**
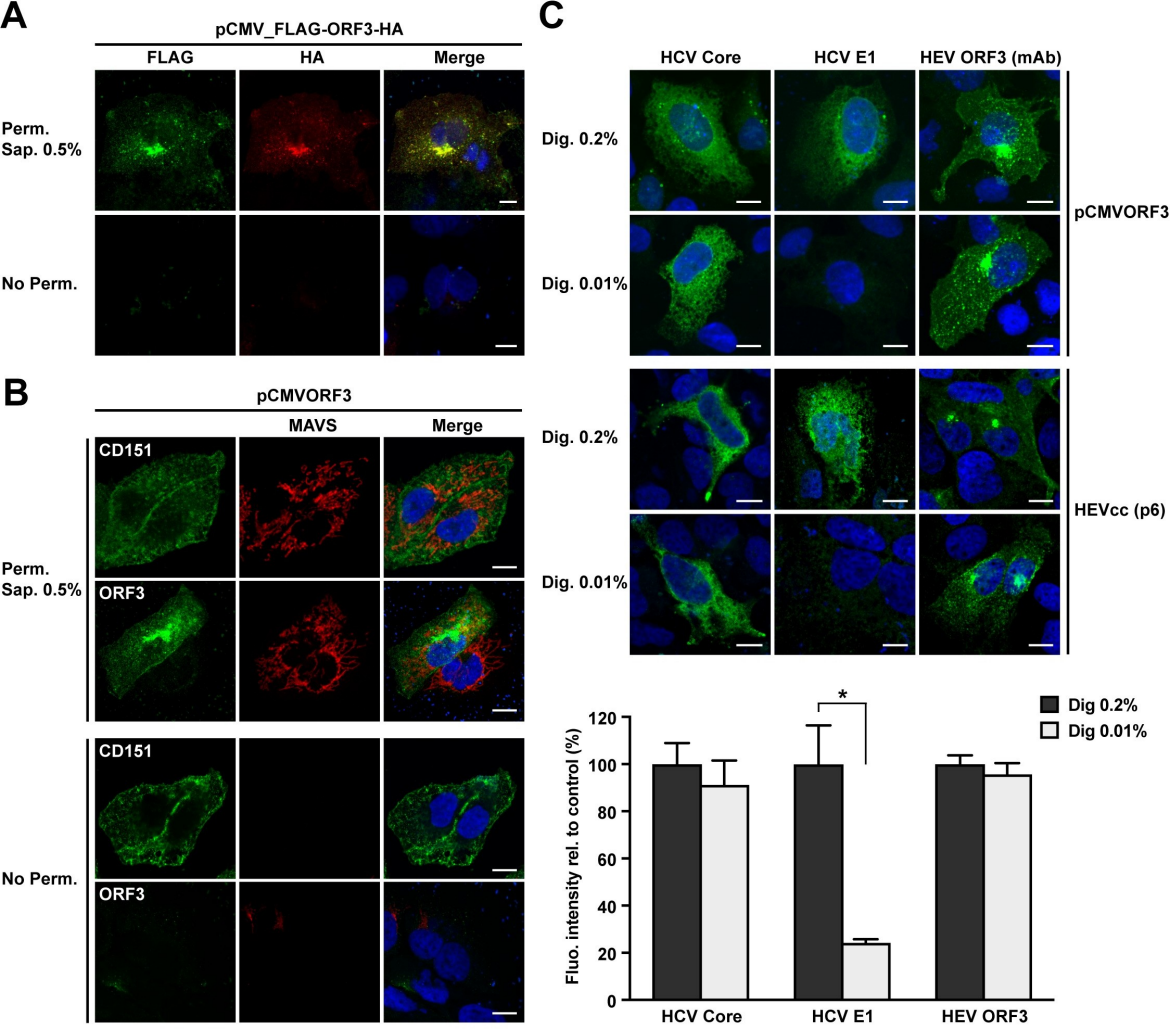
Membrane topology of HEV ORF3 protein. (**A**) N- and C-terminal ends of HEV ORF3 protein are intracellularly exposed. S10-3 cells were transfected with pCMV_FLAG-ORF3-HA and subjected to immunfluorescence detection of HA and FLAG tags, using rabbit pAb anti-HA (Y-11) and mouse mAb anti-FLAG M2, respectively, after permeabilization with 0.5% saponin (Perm. Sap. 0.5%) or in the absence of permeabilization (No Perm.). (**B**) Similarly, S10-3 cells transfected with pCMVORF3 were subjected to immunofluorescence to detect the plasma membrane tetraspanin CD151 (mouse mAb 11G5a), the cytoplasmic protein MAVS (rabbit pAb anti-MAVS) or HEV ORF3 protein using mAb MRB198. Nuclei were stained by DAPI. (**C**) Selective membrane permeabilization. S10-3 cells were transfected with pCMVORF3 or co-transfected with pUHD15-1 and pUHD-Cp7 allowing the expression of the hepatitis C virus (HCV) core-p7 region (top panel) and cultured for 24 h. S10-3 cells were transfected with the HEV p6 infectious clone (middle panel) and cultured for 5 d. All cells were fixed and permeabilized with either 0.2% or 0.01% digitonin. Immunofluorescence detection of the cytoplasmic HCV core with mouse mAb C7-50 or the endoplasmic reticulum luminal HCV E1 glycoprotein with mouse mAb A4 served as controls for selective permeabilization of intracellular membranes. HEV ORF3 protein is detected using anti-ORF3 mAb MRB198. The lower panel shows histograms summarizing fluorescence intensities, as determined by using ImageJ software in 10 to 35 cells per condition, obtained after immunofluorescence with total (Dig. 0.2%) or selective (Dig. 0.01%) membrane permeabilization of S10-3 cells replicating the HEV p6 infectious clone. The asterisk (*) indicates statistically significant results with p<0.001. Scale bars indicate 10 μm.

Palmitoylation occurs on the cytosolic side of the membrane at different subcellular sites depending on the palmitoyltransferase(s) involved. Since the cysteine residues of HEV ORF3 protein are present in the N-terminal segment of the protein, we hypothesized that the N terminus is exposed to the cytosol. To further validate this observation and to position the C-terminal end, we transfected a construct tagged at the N-terminal end with a FLAG epitope and at the C-terminal end with an HA epitope. The tags were detected by immunofluorescence by double staining in the absence of cell permeabilization *vs*. after treatment with 0.5% saponin. As shown in Fig 8A, both epitopes were detectable only after permeabilization of the plasma membrane with saponin. Hence, as suggested by the presence of the palmitoylated cysteines, the N-terminal region of ORF3 protein is exposed to the cytosolic side. Furthermore, these results also suggest that the C-terminal end of the protein is present on the cytosolic side and not exposed to the extracellular space, as it would be the case if HEV ORF3 possessed a transmembrane passage.

Given that the addition of epitope tags may perturb the membrane association of a small protein like HEV ORF3, a similar experiment was performed with the expression of an untagged ORF3 construct (Fig 8B). The detection of the cell surface-expressed tetraspanin CD151 and of cytosolic mitochondrial antiviral-signaling protein (MAVS) have been employed as controls in both conditions, i.e non permeabilized and saponin 0.5% treated cells. Indeed, the cytosolic protein, i.e. MAVS, could be detected only in permeabilized conditions while the surface exposed protein, i.e. CD151, is always accessible to antibody. Thus, we used monoclonal antibody (mAb) MRB198 recognizing an epitope of ORF3 protein in the C-terminal region aa 62–113 to investigate the topology of the viral protein. The ORF3 protein was well detectable at intracellular membranes and, to some extend, at the plasma membrane, by the anti-ORF3 mAb following permeabilization. However, HEV ORF3 protein was not detectable in the absence of permeabilization (Fig 8B). These results further demonstrate that the N- and C-terminal regions of ORF3 protein are both present on the cytosolic side of the plasma membrane.

To corroborate our findings, we also employed selective permeabilization combined with immunofluorescence detection of HEV ORF3 using the specific mAb described above. As described earlier [30], transfection of plasmids allowing the expression of the membrane-associated hepatitis C virus (HCV) proteins core and E1 followed by immunofluorescence detection was used to assess the quality of the selective permeabilization. Indeed, differential digitonin-based permeabilization of cells allows to discriminate between epitopes exposed to the cytosolic *vs*. luminal side of intracellular membranes. Accordingly, we observed the detection of the cytosolically-oriented HCV core protein under total and selective permeabilization conditions, i.e. digitonin 0.2% and 0.01%, respectively (Fig 8C). However, the luminal HCV E1 glycoprotein was detectable only following total permeabilization. Detection of HEV ORF3 protein was carried out with recombinant mAb MRB198 under both permeabilization conditions with a similar pattern of subcellular localization, i.e. at the plasma and intracellular membranes (Fig 8C). The lack of difference between the detection of ORF3 protein under the two permeabilization conditions indicates that the epitope recognized by the mAb is oriented toward the cytosol. Importantly, given that these data were obtained under ORF3 single expression settings, we confirmed our findings using the same approach in S10-3 cells replicating the full-length HEV infectious clone p6 for which fluorescence intensity was measured and plotted for the different conditions (Fig 8C).

Altogether, our results indicate that HEV ORF3 protein is exposed to the cytosolic side of the plasma membrane as well as of the intracellular membranes. Thus, our data indicate that HEV ORF3 protein is membrane-associated *via* palmitoylation of the N-terminal segment and does not traverse the membrane.

## Discussion

In the present study, we identified the N-terminal region of HEV ORF3 protein as the determinant for membrane association and oligomerization. More precisely, we found that ORF3 protein is palmitoylated at the N-terminal cysteine-rich segment. Palmitoylation of HEV ORF3 protein determines its membrane association, subcellular localization and likely stabilizes the viral protein. Moreover, the mutation of cysteine residues of ORF3 protein engaged in palmitoylation strongly impairs the secretion of infectious HEV. Additionally, investigation of the membrane topology of HEV ORF3 protein by selective permeabilization coupled with immunofluorescence detection supports a model where the entire ORF3 protein is oriented toward the cytosolic side of the membrane.

We have found that ORF3 protein is membrane-associated and localized to intracellular membranes as well as the plasma membrane. While the latter localization is not predominant in HEV-replicating S10-3 cells, ORF3 protein accumulates more importantly at this compartment when expressed alone in U-2 OS or Hep293TT (S1D Fig). Inhibition by 2-BP revealed that subcellular localization of ORF3 protein is determined by palmitoylation. Similarly, it has been shown that the NB glycoprotein of influenza B virus is palmitoylated and that this posttranslational modification determines the subcellular localization of the viral protein to the plasma membrane [31]. Emerson and colleagues reported an accumulation of ORF3 protein at the apical membrane of C25j cells, a subclone of the human intestinal cell line Caco-2 which was associated with a preferential release of infectious virus at this apical membrane [19]. Furthermore, it is interesting to note that ORF3 protein is present at bile canaliculi of humanized liver chimeric mice infected with HEV [32, 33]. Therefore, these observations are in agreement with a plasma membrane and bile canalicular localization of the ORF3 protein.

Oligomerization of HEV ORF3 protein had been previously investigated in yeast 2-hybrid system and revealed that determinants for self-interaction reside in aa segment 71–113 [25]. A recent report showed oligomerization of ORF3 protein in mammalian cells by co-immunoprecipitation [23]. In our study, we confirm oligomerization of ORF3 protein by co-immunoprecipitation as well as FRET and we map the interacting domain to the region spanning aa 1 to 53. However, our results cannot exclude a possible interaction domain in segment aa 71–113 as suggested by Tyagi and colleagues [25]. The N-terminal segment comprises the palmitoylated cysteine-rich domain as well as the following hydrophobic domain aa 28–53. Palmitoylation is known to regulate protein-protein interactions [34]. Therefore, it is likely that the C16 acyl chains linked to the cysteine residues are important contributors of ORF3 oligomerization, possibly together with the C-terminal region of the protein. Indeed, similarly to observations made with the oligomeric HCV nonstructural protein 4B [35], protein oligomerization may depend on several interacting domains.

While ORF3 is often referred to as a 13-kDa protein, reports in the literature showed ORF3 protein with higher apparent MW at about 15 kDa in infected cells [21, 22, 36] as well as *in vivo* in liver chimeric mice infected with HEV [32]. Discrimination of the MW of HEV ORF3 protein required optimal separation by high percentage SDS-PAGE. Of note, we have included the ORF3_S70A_ mutant in our analyses to examine the potential contribution of the phosphorylation described previously [24]. However, our data shows that alanine substitution at aa position 70 does not change the migration pattern of ORF3 protein in SDS-PAGE. Thus, our analyses did not allow to state whether HEV ORF3 protein is phosphorylated in addition to its palmitoylation.

While the HEV ORF3 sequence of Orthohepeviruses A, including HEV gt 1 and gt 3, is not well conserved as compared to the one of Orthohepeviruses B or C, i.e. avian or rat HEV, the presence of cysteine residues in the N-terminal region is conserved. Interestingly, avian HEV ORF3 protein, which possesses 10 cysteine residues among its N-terminal 30 aa (S4A Fig), displays a similar subcellular localization as compared to HEV genotype 3 ORF3 protein (S4B Fig). Moreover, avian HEV ORF3 protein, which displays an apparent molecular weight above the theoretical one (15 kDa *vs*. 11–12 kDa), can incorporate ^3^H-palmitate (S4C Fig) when expressed in human cells. These observations strongly suggest that palmitoylation of the HEV ORF3 protein is a conserved feature among the Orthohepeviruses.

A recent report by Ding *et al*. suggested that ORF3 protein possesses an ion channel activity required for virus secretion [23]. Such function implies that ORF3 protein has a transmembrane segment to form a pore upon oligomerization. As shown here, the protein sequence of HEV ORF3 is highly conserved in the N-terminal region and more specifically in region aa 1–28. A transmembrane segment is predicted by some algorithms (i.e. PHDHTM, TMpred, TMHMM) in region aa 31–52, however with less consistency for HEV ORF3 protein of genotype 4. In addition, secondary structure prediction for an alpha-helix is poor (Fig 1A). Furthermore, we experimentally observed that ORF3 expression constructs where the predicted transmembrane segment (aa 28–53) alone or the C-terminal part (aa 28–113) is fused to GFP were found to be mainly soluble in transfected cells (Fig 3). As it is difficult to prove the existence of an internal transmembrane segment when expressed out of its natural protein context, we employed an antibody accessibility assay using selective membrane permeabilization and immunofluorescence with a mAb against the C-terminal part (epitope aa 62–113) of ORF3 protein. Our data demonstrate that the epitope present in the C-terminal part of HEV ORF3 protein is detectable upon selective permeabilization of the plasma membrane, indicating its presence on the cytosolic side of cellular membranes. Given the palmitoylation of ORF3 protein at N-terminal cysteine-rich domain (aa 5–21), our data reveal that ORF3 protein is entirely oriented to the cytosolic side while associated with the membrane at least through palmitoylation. Supporting our findings, the well described PSAP motif of HEV ORF3 protein (aa 95–99 for genotype 3) required for the interaction with Tsg101 [19, 21, 37] needs to be oriented toward the cytosol to allow association with the ESCRT-I protein. Furthermore, ORF3 protein is not only present in infected cells but is also believed to be part of the quasi-enveloped virus secreted into the bloodstream or in cell culture [15, 19, 22]. These reports demonstrated that ORF3 protein, more precisely epitope aa 90–113, is detectable only when quasi-enveloped virus is treated with a detergent to permeabilize the membrane. Hence, consistent with our findings, these reports indicate that ORF3 protein is present inside the exosomes constituting the quasi-enveloped HEV particle [16]. Thus, the membrane topology suggested by Ding and colleagues, where ORF3 protein would possess a transmembrane segment comprising aa 30–52, is not fully supported by our observations as well as by others. However, viral proteins are often multifunctional and may adopt different membrane topologies to exert different functions, as it is the case for the hepatitis B virus large surface protein [38], the fusion protein of Newcastle disease virus [39] or HCV nonstructural protein 4B [30]. Therefore, it is conceivable that a subset of ORF3 protein at a particular localization or at a defined stage of the infection forms pores to exert ion channel activity.

Previous reports showed that HEV ORF3 protein is crucial for infectious virus secretion [20] and is incorporated into the quasi-enveloped virion [15, 22]. Given the membrane association as well as the palmitoylation of ORF3 protein described here, one may hypothesize that the posttranslational modification of ORF3 protein regulates its function in virus secretion. Indeed, we demonstrated that the cysteine residues of HEV ORF3 protein, engaged in palmitoylation, are essential for the secretion of infectious particles. Interestingly, a recent report demonstrated that palmitoylation of the small transframe protein of Sindbis virus, another positive-strand RNA virus, determines its localization to the plasma membrane and regulates its incorporation into the particle and thereby virus secretion [40]. Hence, future investigations shall aim at identifying the host palmitoyltransferase(s) responsible for ORF3 protein modification to further explore the functional consequences of this posttranslational modification on the virus life cycle but also the pathogenesis of HEV.

To conclude, our results point toward an important host-dependent posttranslational modification of HEV ORF3 protein, its palmitoylation, which has important functional implications for HEV biology.

## Materials and methods

### Cell lines and reagents

U-2 OS human osteosarcoma (obtained from the American Type Culture Collection, Rockville, MD) and S10-3 human hepatocellular carcinoma cell lines (kindly provided by Suzanne U. Emerson, National Institutes of Health, Bethesda, MD) were maintained in Dulbecco’s modified Eagle medium supplemented with 10% fetal bovine serum. The Hep293TT human hepatoblastoma cell line (kindly provided by Gail E. Tomlinson, San Antonio, TX) was maintained in Roswell Park Memorial Institute medium containing HEPES buffer, L-glutamine and 10% fetal bovine serum. Plasmids encoding the full-length HEV infectious clones p6 (Kernow-C1 strain) [41] and pSK-HEV-2 (Sar55 strain) [42] as well as the subgenomic replicon p6-luc were kindly provided by Suzanne U. Emerson. The polymerase-deficient replicon construct p6-luc-GAD was prepared as described previously [1, 43]. Plasmid encoding the full-length HEV infectious clone 83–2 [44] was kindly provided by Koji Ishii as well as Takaji Wakita (Department of Virology II, National Institute of Infectious Diseases, Japan).

### Antibodies

Monoclonal antibodies (mAb) anti-FLAG M2 and anti-β-actin were from Sigma-Aldrich (Saint-Louis, MI). Polyclonal antibody (pAb) against HA tag (Y-11) and mouse mAbs 11G5a against CD151 were from Santa Cruz Biotechnology (Dallas, TX). Rabbit pAb against MAVS was from Enzo Life Sciences (Farmingdale, NY). MAb JL8 against GFP was from Takara Bio (Mountain View, CA), mAb G1/296 against CLIMP-63 from Enzo Life Sciences and mAb JG1 against heat shock protein 70 (Hsp70) from Affinity Bioreagents (Golden, CO). Secondary antibodies were HRP-conjugated anti-mouse (GE Healthcare; Chicago, IL) and anti-rabbit (Agilent Technologies; Santa Clara, CA). Rabbit anti-ORF3 and swine anti-ORF2 pAbs were kind gifts from Suzanne U. Emerson. Rabbit antiserum against HEV ORF2 was a kind gift from Rainer G. Ulrich (Friedrich-Loeffler-Institute, Greifswald-Insel Riems, Germany) [45]. Recombinant mouse mAb against genotype 3 HEV ORF3 protein was produced as described [46] by the Geneva Antibody Facility (https://web.expasy.org/abcd/ABCD_RB198). MAbs C7-50 against HCV core [47] and A4 against HCV E1 [48] (kindly provided by Jean Dubuisson, University of Lille, France) have been described.

### Plasmids

Primers used in this study are listed in S1 Table. Unless specified otherwise, the constructs described here were derived from the HEV genotype 3 p6 infectious clone (Kernow-C1 strain, GenBank accession number JQ679013) as template and verified by sequencing.

ORF3 expression construct pCMVORF3 and pCMVORF3_Δ19_ were prepared by PCR amplification with primers ORF3-1-Hind-fd or ORF3-19-Hind-fd, respectively, and ORF3-113st-Bam-rv, followed by cloning into the *Hind*III/*Bam*HI sites of pcDNA3.1(+) (Thermo Fischer Scientific, Waltham, MA). The phosphorylation-deficient mutant ORF3 construct pCMVORF3_S70A_ was prepared by site-directed mutagenesis using primer pair HEVKc1_S70A-fd and HEVKc1_S70A-rv as well as pCMVORF3 as template. Similarly, pCMVORF3_gt1, allowing the expression of genotype 1 HEV ORF3 protein, was prepared by PCR amplification with primers ORF3-gt1-1Hind-fd and ORF3-gt1-115Bam-rv, using pSK-HEV-2 (Sar55 strain, GenBank accession number AF444002) as template, followed by cloning into the *Hind*III/*Bam*HI sites of pcDNA3.1(+).

Constructs used for co-immunoprecipitation, i.e. pCMVORF3-FLAG and pCMVORF3-HA, were prepared by PCR amplification with forward primer ORF3-1-Hind-fd and reverse primer ORF3-113-Bam-rv, followed by *Hind*III/*Bam*HI digestion and cloning into previously described plasmids pCMV-X-FLAG and pCMVNS4B-HA [35]. The double-tagged ORF3 construct pCMVFLAG-ORF3-HA used for selective permeabilization was prepared by PCR amplification with forward primer ORF3noATG-1-Bsp-fd and reverse primer BGH-rv, followed by *Bsp*EI/*Bam*HI digestion and cloning into pCMVFLAG-JFH4B-HA [35].

GFP fusion constructs pCMVORF3-GFP, pCMVORF3_1-53_-GFP, pCMVORF3_1-28_-GFP, pCMVORF3_28-113_-GFP, pCMVORF3_53-113_-GFP and pCMVORF3_28-53_-GFP were prepared by PCR amplification with forward primers ORF3-1-Hind-fd, ORF3-28-Hind-fd or ORF3-53-Hind-fd and reverse primer ORF3-113-Bam-rv, ORF3-53-Bam-rv or ORF3-28-Bam-rv, followed by cloning into the *Hind*III/*Bam*HI sites of pCMVKEB-GFP [49].

FRET constructs, i.e. pCMVORF3-CFP, pCMVORF3-YFP, pCMVORF3_1-93_-YFP, pCMVORF3_1-93_-CFP, pCMVORF3_1-70_-CFP, pCMVORF3_1-70_-YFP, pCMVORF3_1-53_-CFP, pCMVORF3_1-53_-YFP, pCMVORF3_28-113_-CFP, pCMVORF3_28-113_-YFP, pCMVORF3_53-113_-CFP and pCMVORF3_53-113_-YFP, were prepared by PCR amplification with forward primers ORF3-1-Hind-fd, ORF3-28-Hind-fd or ORF3-53-Hind-fd and reverse primer ORF3-113-Bam-rv, ORF3-93-Bam-rv, ORF3-70-Bam-rv or ORF3-53-Bam-rv, followed by cloning into the *Hind*III/*Bam*HI sites of pCMVNS4B-YFP or pCMVNS4B-CFP [35].

Alanine substitution mutants, i.e. ORF3_C1-4_, ORF3_C5-8_ and ORF3_C1-8_, were synthesized by GenScript (Piscataway, NJ). Constructs pCMVORF3_C1-4_, pCMVORF3_C5-8_ and pCMVORF3_C1-8_ were prepared by cassette exchange by cloning into *Hind*III-*Bam*HI sites of pCMVORF3. GFP fusion constructs harboring the mutations, i.e. pCMVORF3_C1-4_-GFP, pCMVORF3_C5-8_-GFP and pCMVORF3_C1-8_-GFP, were prepared by PCR amplification from the corresponding pCMVORF3 constructs with forward primer CMV-fd and reverse primer ORF3-113-Bam-rv, followed by *Hind*III/*Bam*HI digestion and cloning into pCMVKEB-GFP.

The construct allowing expression of ORF3 in a wheat germ-based cell free expression system, pEU-ORF3, was prepared by cassette exchange of, the ORF3 coding sequence by digestion of pCMVORF3 with *Pme*I-*Bam*HI and cloning into the *Eco*RV-*Bam*HI sites of pEU-MCS-E01 (CellFree Sciences; Matsuyama, Japan).

A 977-bp DNA fragment of the HEV p6 clone bordered by *Afl*II and *Pml*I restriction sites and harboring a serine substitution of 5 N-terminal cysteine residues, i.e. p6_C_5_S, was synthesized by GenScript. Construct p6_C_5_S was prepared by cloning into the *Afl*II-*Pml*I sites of the p6 wt plasmid. ORF3 expression constructs harboring the C_5_S mutation, i.e. pCMVORF3_C5S_ and pCMVORF3_C5S_-GFP, were prepared by PCR amplification from the p6_C_5_S construct with forward primer ORF3-1-Hind-fd and reverse primer ORF3-113st-Bam-rv or ORF3-113-Bam-rv, respectively, followed by *Hind*III/*Bam*HI digestion and cloning into pcDNA3.1(+) or pCMVKEB-GFP.

### Immunoblot

Protein lysates were prepared and subjected to sodium dodecyl sulfate-polyacrylamide gel electrophoresis (SDS-PAGE), followed by immunoblot analysis as described previously [50].

### *In vitro* transcription, electroporation and infection assays

Capped full-length HEV RNA was produced by *in vitro* transcription using the mMACHINE mMESSAGE Kit from Ambion (Thermo Fischer Scientific), followed by electroporation into Hep293TT cells or transfection of S10-3 cells using TransIT-mRNA transfection kit (Mirus Bio LLC, Madison, WI) [43]. Cells were cultured for 5 to 8 days before harvesting to prepare protein lysates, to perform immunofluorescence or to collect intra- and extracellular compartments for infectivity determination.

### Fluorescence resonance energy transfer (FRET)

FRET analysis has been performed as previously described [35]. Briefly, U-2 OS cells cultured on glass coverslips were transfected with constructs expressing CFP- and YFP-tagged proteins and fixed at 24 h posttransfection with 2% paraformaldehyde for 5 min and the coverslips were mounted on glass slides with SlowFade reagent (Thermo Fischer Scientific). Acceptor photobleaching FRET was performed using an SP5 AOBS confocal laser scanning microscope (Leica; Wetzlar, Germany).

### Radiolabeling and immunoprecipitation

To detect palmitoylation, U-2 OS cells were transfected with pCMVORF3, pCMVORF3_C1-4_ or pCMVORF3_C5-8_ plasmids and Hep293TT were electroporated with full-length HEV p6 or 83–2 RNAs prior to incubation with radiolabelled palmitate 24 h or 6 days post-transfection, respectively. The cells were washed twice and incubated at 37°C for 1 h in the respective medium without FCS then followed by 3 h incubation in the same medium supplemented with 200 μCi/mL ^3^H-palmitate (9,10-^3^H(N)) (American Radiolabeled Chemicals, St Louis, MO). After three washes with cold PBS, cells were lysed in immunoprecipitation buffer (50 mM Tris-HCl pH 7.4; 1 mM EDTA; 150 mM NaCl; 1% Triton X-100) supplemented with 1X cOmplete protease inhibitor cocktail (Roche, Basel, Switzerland). Immunoprecipitation was carried out by the incubation of each protein lysates overnight at 4°C with 50 μL of Dynabeads Protein G (Thermo Fischer Scientific) pre-adsorbed with 1 μl of rabbit anti-ORF3 pAb, gift from Suzanne Emerson (NIH, Bethesda, MD), or unrelated rabbit serum as control, following manufacturers’s recommendations. After three washes with 0.02% Tween-20 in PBS using a magnetic device, elution was performed by incubation of the beads in 30 μL of 100 mM glycine pH 3 for 5 min at RT. Samples were then incubated for 5 min at 90°C in Laemmli buffer and separated onto a 17% SDS-PAGE. Gel was either incubated 30 min at 20°C in a fixative solution (25% isopropanol, 65% H_2_O, 10% acetic acid), followed by a 30 min incubation with signal enhancer Amplify NAMP100 (GE Healthcare) and subjected to autoradiography for 40 days or undergone immunoblot analysis with pAb anti-ORF3.

### Confocal microscopy

S10-3 cells were grown onto 22-mm diameter glass coverslips before being assayed. Briefly, cells were fixed 10 min with 2% paraformaldehyde at 20°C. Fixed cells were washed with PBS and permeabilized with 0.5% saponin in PBS for 5 min, followed by a 20-min incubation in 10% goat serum blocking solution. Cells were successively incubated for 1 h with the first antibody and then with Alexa Fluor 488- and 594-conjugated anti-mouse and anti-rabbit IgG antibodies (Thermo Fisher Scientific). Additional incubation with DAPI (Thermo Fisher Scientific) allowed staining of the nuclei. Slides were prepared using antifade mounting medium ProLong (Thermo Fisher Scientific). Cells were examined under a Zeiss LSM 710 quasar laser scanning fluorescence confocal microscope and images were treated with ImageJ software.

### Selective permeabilization

S10-3 cells were seeded onto glass coverslips and transfected 24 h later with ORF3 expression vectors or co-transfected with pUHD15-1 [51] and pUHD-Cp7 plasmids allowing the expression of HCV core-p7 region, as described previously [30]. Forty-eight h post-transfection cells were fixed with 2% paraformaldehyde (Sigma-Aldrich) for 10 min and then permeabilized, totally, with either 0.5% saponin or 0.2% digitonin (Sigma-Aldrich) or selectively with 0.01% digitonin. Cells were then washed and incubated 15 min at 20°C in blocking buffer containing 3% bovine serum albumin in PBS. Indirect immunofluorescence was then performed by one-hour incubation at 20°C with primary antibody, followed by 3 PBS washes and incubation with the secondary antibody as described above.

### Statistical analyses

Significance values were calculated by using the unpaired t test with the GraphPad Prism 6 software package (GraphPad Software).

## Supporting information

S1 Table Oligonucleotide sequences. Sequences of the primers used to prepare the constructs employed in this study are given in the 5’ to 3’ orientation.
(DOCX)

S1 Fig Immunoblot analyses of different ORF3 constructs. (**A**) Immunoblot analysis of GFP-fused ORF3 and deletion constructs. U-2 OS cells were transiently transfected with pCMVORF3-GFP (1–113), pCMVORF3_1-28-GFP (1–28), pCMVORF3_1-53-GFP (1–53), pCMVORF3_28-113-GFP (28–113), pCMVORF3_53-113-GFP (53–113), pCMVORF3_28-53-GFP (28–153) as well as with pCMV-GFP (GFP). Cell lysates prepared 24 h post-transfection were separated by 12% SDS-PAGE followed by immunoblot analysis using JL8 mAb against GFP. Non-transfected cells (-) served as control. (**B**) Immunoblot analysis of ORF3 mutants. U-2 OS cells were transfected with pCMVORF3, pCMVORF3_S70A_ or pCMVORF3_Δ19_. Protein lysates obtained 24 h post-transfection as well as ORF3 protein expressed using wheat germ extract (WG) were separated by 17% SDS-PAGE and subjected to immunoblot with anti-ORF3 pAb. (**C**) Immunoblot analyses of ORF3 genotype 1. S10-3 cells were transiently transfected with pCMVORF3 (gt3) and pCMVORF3_gt1 (gt1), and separated, together with a wheat germ expressed ORF3 protein (WG) sample, by 17% SDS-PAGE followed by immunoblot analysis using pAb anti-ORF3. Non-transfected cells (-) served as control. (**D**) Subcellular localization of HEV ORF3 protein in different cell lines. S10-3, U-2 OS or Hep293TT cells were transfected with pCMVORF3. Cells were fixed 48 h post-transfection and analyzed by fluorescence microscopy after immunofluorescence staining of HEV ORF3 protein using anti-ORF3 rabbit pAb. Scale bars indicate 10 μm. (**E**) Immunoblot analysis of single alanine substitution of the cysteine residues of ORF3 protein. U-2 OS cells were transiently transfected with pCMVORF3 (wt), pCMVORF3_C5A_, pCMVORF3_C11A_, pCMVORF3_C12A_, pCMVORF3_C13A_, pCMVORF3_C16A_, pCMVORF3_C18A_, pCMVORF3_C20A_ and pCMVORF3_C21A_. Cell lysates prepared 24 h post-transfection were separated by 17% SDS-PAGE followed by immunoblot analysis using anti-ORF3 pAb. Non-transfected cells (-) served as control. (**F**) Immunoblot analysis of GFP-ORF3 mutants constructs. U-2 OS cells were transiently transfected with pCMVORF3-GFP (wt), pCMVORF3_C1-4_-GFP, pCMVORF3_C5-8_-GFP, pCMVORF3_C1-8_-GFP and pCMV-GFP. Cell lysates prepared 24 h post-transfection were separated by 12% SDS-PAGE followed by immunoblot analysis using JL8 mAb against GFP. (**G**) Immunoblot analysis of FLAG-ORF3-HA fusion construct. U-2 OS cells were transiently transfected with pCMVFLAG-ORF3-HA. Cell lysate obtained 24 h post-transfection was separated by 17% SDS-PAGE followed by immunoblot analysis using either anti-HA (Y-11) pAb or anti-FLAG M2 mAb.
(TIF)

S2 Fig Hep293TT human hepatoblastoma cells support HEV RNA replication and infectious particle production. (**A**) Hep293TT cells are replicating HEV subgenomic replicon. S10-3 and Hep293TT were transfected with p6-luc HEV replicon and the cell culture medium was harvested every day to measure the gaussia luciferase activity. S10-3 cells transfected with the polymerase-deficient construct p6-luc-GAD served as negative control (Neg.). (**B**) Hep293TT cells can produce infectious HEV particle. S10-3 and Hep293TT cells were transfected with full-length p6 HEV RNA. Five days post-transfection, culture supernatants were harvested and cell lysates were prepared by freeze-and-thaw cycles followed by clarification by centrifugation at 2,000 g for 15 min Intracellular (Intra) and extracellular (Extra) infectivities were determined by foci forming assay with HepG2/C3A cells using respectively, the cell lysates and the culture supernatants as inoculum. Immunofluorescence detection of the capsid protein was performed with mAb 1E6 against HEV ORF2. ffu: focus forming unit.
(TIF)

S3 Fig Treatment with 2-bromopalmitate (2-BP) partially inhibits the posttranslational modification of HEV ORF3 protein. U-2 OS cells transfected with pCMVORF3 and cultured in presence of 5% FCS and with increasing concentrations of 2-BP were harvested 24 h post-transfection. Immunoblot analysis was done with pAb anti-ORF3. Corresponding lower *vs* upper band intensity ratio is shown below the immunoblot.
(TIF)

S4 Fig Postranslational modification of ORF3 protein is conserved in other Orthohepevirus. (**A**) Amino acid sequences of ORF3 from HEV genotype 3 (HEV3) (GenBank accession number AB740232) and avian HEV (GenBank accession number AY535004) were aligned by ClustalW. The degree of aa physicochemical conservation at each position is shown on the bottom line and can be inferred with the similarity index according to ClustalW convention (asterisk, invariant; colon, highly similar; dot, similar) [52]. (**B**) Subcellular localization of avian HEV ORF3. U-2 OS cells transfected with pCMVORF3-FLAG or pCMVORF3_avian_-FLAG were subjected to immunofluorescence using anti-FLAG M2 mAb and DAPI staining of the nucleus before confocal microscopy analysis. Scale bars indicate 10 μm. (**C**) S10-3 cells transfected with pCMVORF3-FLAG or pCMVORF3_avian_-FLAG were incubated with Dulbecco Modified Eagle Medium‎ supplemented with ^3^H-palmitate for 3 h. Protein lysates were prepared and subjected to imunoprecipitation with either anti-FLAG M2 mAb (+) or non-relevant mouse mAb (-). After immunoprecipitation, the elution samples were separated by 17% SDS-PAGE and subjected to either immunoblot with anti-FLAG M2 mAb followed by chemiluminescence revelation or autoradiography (40 days of exposure).
(TIF)

S5 Fig Analysis of mutant C5S expressed as a GFP fusion protein or in the context of full-length HEV RNA. (**A**) S10-3 cells transfected with pCMVORF3-GFP (wt) or pCMVORF3_C5S_-GFP (C5S) were analyzed 48 h post-transfection by 12% SDS-PAGE followed by immunoblot with JL8 mAb against GFP. (**B**) Naïve Hep293TT cells (-) or Hep293TT cells replicating the full-length p6 or p6_C5S HEV RNA were lysed 6 d post-electroporation, followed by 17% SDS-PAGE and immunoblot with either anti-ORF3 pAb, anti-ORF2 mAb or anti-β-actin mAb.
(TIF)
