# Supplementary material for: Palmitoylation mediates membrane association of hepatitis E virus ORF3 protein and is required for infectious particle secretion
Source: PLoS Pathog. 2018 Dec 10;14(12):e1007471. doi: 10.1371/journal.ppat.1007471 (PMC6307819; doi:10.1371/journal.ppat.1007471)
Supplement: S1 Table — Sequences of the primers used to prepare the constructs employed in this study are given in the 5’ to 3’ orientation. (DOCX) [file ppat.1007471.s001.docx]

**S1 Table. Oligonucleotide sequences.**

| Name | Sequence (5’-3’) |
| --- | --- |
| ORF3-1-Hind-fd | ATGATGAAGCTTACCATGGGATCACCATGTGCCCTA |
| ORF3-113st-Bam-rv | ATGATGGGATCCTCAACGGCGCAGCCCCAGCTGGG |
| ORF3-gt1-1Hind-fd | ATGATGAAGCTTACCATGGGTTCGCGACCATGCGCCCT |
| ORF3-gt1-115Bam-rv | ATGATGGATCCTTAGCGGCGCGGCCCCAGCTGTGGTA |
| HEVKc1_S70A-fd | CCTTCGCCGCCGATAGCGTTTCACAATCCGG |
| HEVKc1_S70A-rv | CCGGATTGTGAAACGCTATCGGCGGCGAAGG |
| ORF3-28-Hind-fd | ATGATGAAGCTTACCATGAGCCGTCTGGCCGTCGTCGT |
| ORF3-53-Hind-fd | ATGATGAAGCTTACCATGAGCCCTTCGCCCTCCCCTAT |
| ORF3-113-Bam-rv | ATGATGGGATCCACGGCGCAGCCCCAGCTGGG |
| ORF3-28-Bam-rv | ATGATGGGATCCGCTGGCCGGCCGGTGGCGC |
| ORF3-53-Bam-rv | ATGATGGGATCCGCTGAGAATCAACCCTGTCA |
| ORF3-70-Bam-rv | ATGATGGGATCCCGATATCGGCGGCGAAGGGGT |
| ORF3-94-Bam-rv | ATGATGGGATCCACTGGTCACGCCAAGCGGAGC |
| ORF3-19-Hind-fd | TTTAAACTTAAGCTTACCATGCTATGCTGCCCGCGCCACCG |
| ORF3noATG-1-Bsp-fd | ATAAGTCCGGAGGATCACCATGTGCCCTAGG |
| BGH-rv | TAGAAGGCACAGTCGAGG |
| CMV-fd | CGCAAATGGGCGGTAGGCGTG |
