# Supplementary figures and images for: Palmitoylation mediates membrane association of hepatitis E virus ORF3 protein and is required for infectious particle secretion
Source: PLoS Pathog. 2018 Dec 10;14(12):e1007471. doi: 10.1371/journal.ppat.1007471 (PMC6307819; doi:10.1371/journal.ppat.1007471)

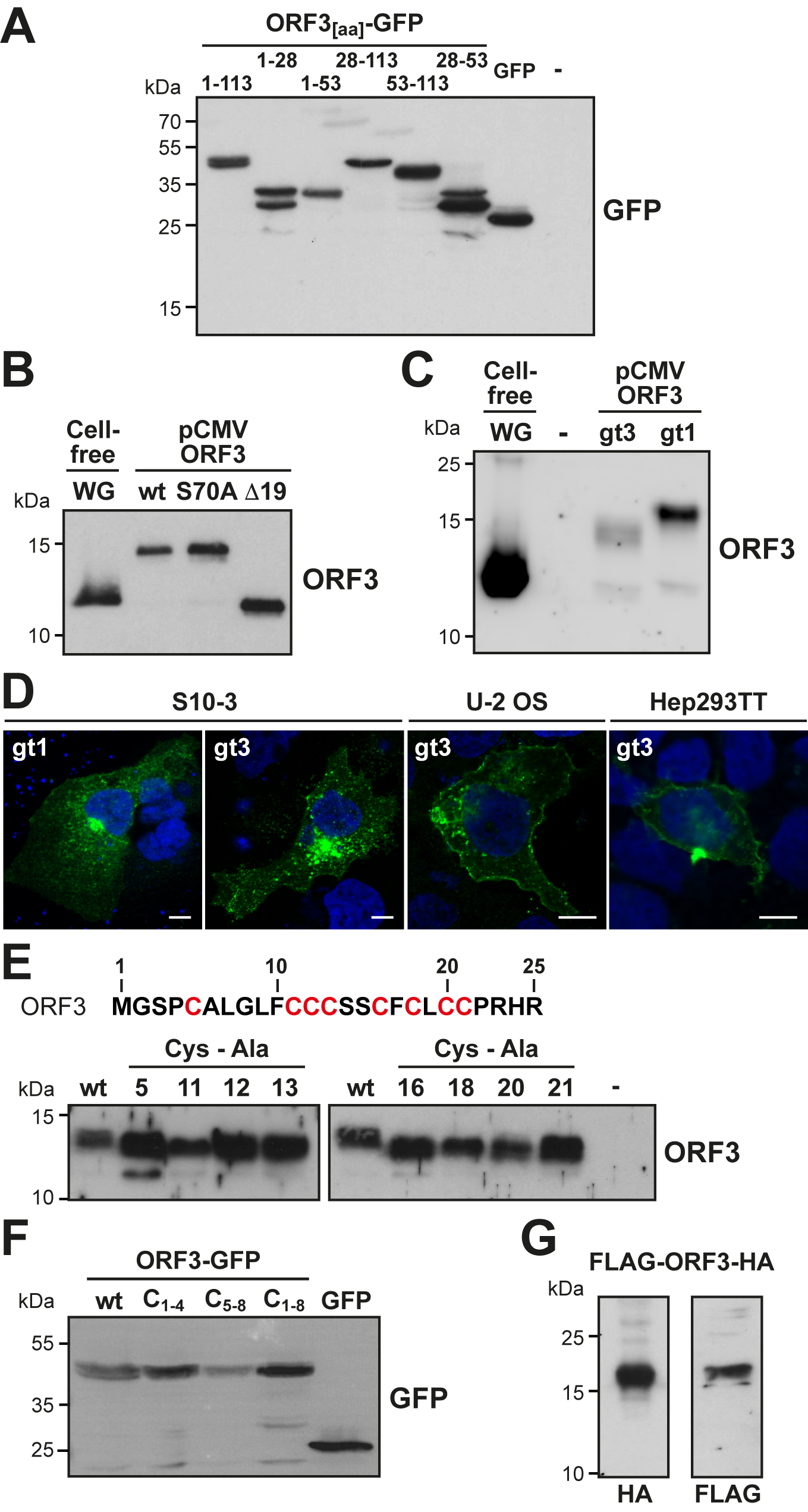

Supplement: S1 Fig — (A) Immunoblot analysis of GFP-fused ORF3 and deletion constructs. U-2 OS cells were transiently transfected with pCMVORF3-GFP (1–113), pCMVORF3_1-28-GFP (1–28), pCMVORF3_1-53-GFP (1–53), pCMVORF3_28-113-GFP (28–113), pCMVORF3_53-113-GFP (53–113), pCMVORF3_28-53-GFP (28–153) as well as with pCMV-GFP (GFP). Cell lysates prepared 24 h post-transfection were separated by 12% SDS-PAGE followed by immunoblot analysis using JL8 mAb against GFP. Non-transfected cells (-) served as control. (B) Immunoblot analysis of ORF3 mutants. U-2 OS cells were transfected with pCMVORF3, pCMVORF3S70A or pCMVORF3Δ19. Protein lysates obtained 24 h post-transfection as well as ORF3 protein expressed using wheat germ extract (WG) were separated by 17% SDS-PAGE and subjected to immunoblot with anti-ORF3 pAb. (C) Immunoblot analyses of ORF3 genotype 1. S10-3 cells were transiently transfected with pCMVORF3 (gt3) and pCMVORF3_gt1 (gt1), and separated, together with a wheat germ expressed ORF3 protein (WG) sample, by 17% SDS-PAGE followed by immunoblot analysis using pAb anti-ORF3. Non-transfected cells (-) served as control. (D) Subcellular localization of HEV ORF3 protein in different cell lines. S10-3, U-2 OS or Hep293TT cells were transfected with pCMVORF3. Cells were fixed 48 h post-transfection and analyzed by fluorescence microscopy after immunofluorescence staining of HEV ORF3 protein using anti-ORF3 rabbit pAb. Scale bars indicate 10 μm. (E) Immunoblot analysis of single alanine substitution of the cysteine residues of ORF3 protein. U-2 OS cells were transiently transfected with pCMVORF3 (wt), pCMVORF3C5A, pCMVORF3C11A, pCMVORF3C12A, pCMVORF3C13A, pCMVORF3C16A, pCMVORF3C18A, pCMVORF3C20A and pCMVORF3C21A. Cell lysates prepared 24 h post-transfection were separated by 17% SDS-PAGE followed by immunoblot analysis using anti-ORF3 pAb. Non-transfected cells (-) served as control. (F) Immunoblot analysis of GFP-ORF3 mutants constructs. U-2 OS cells were transiently transfected with pCMVORF3 [file ppat.1007471.s002.tif]

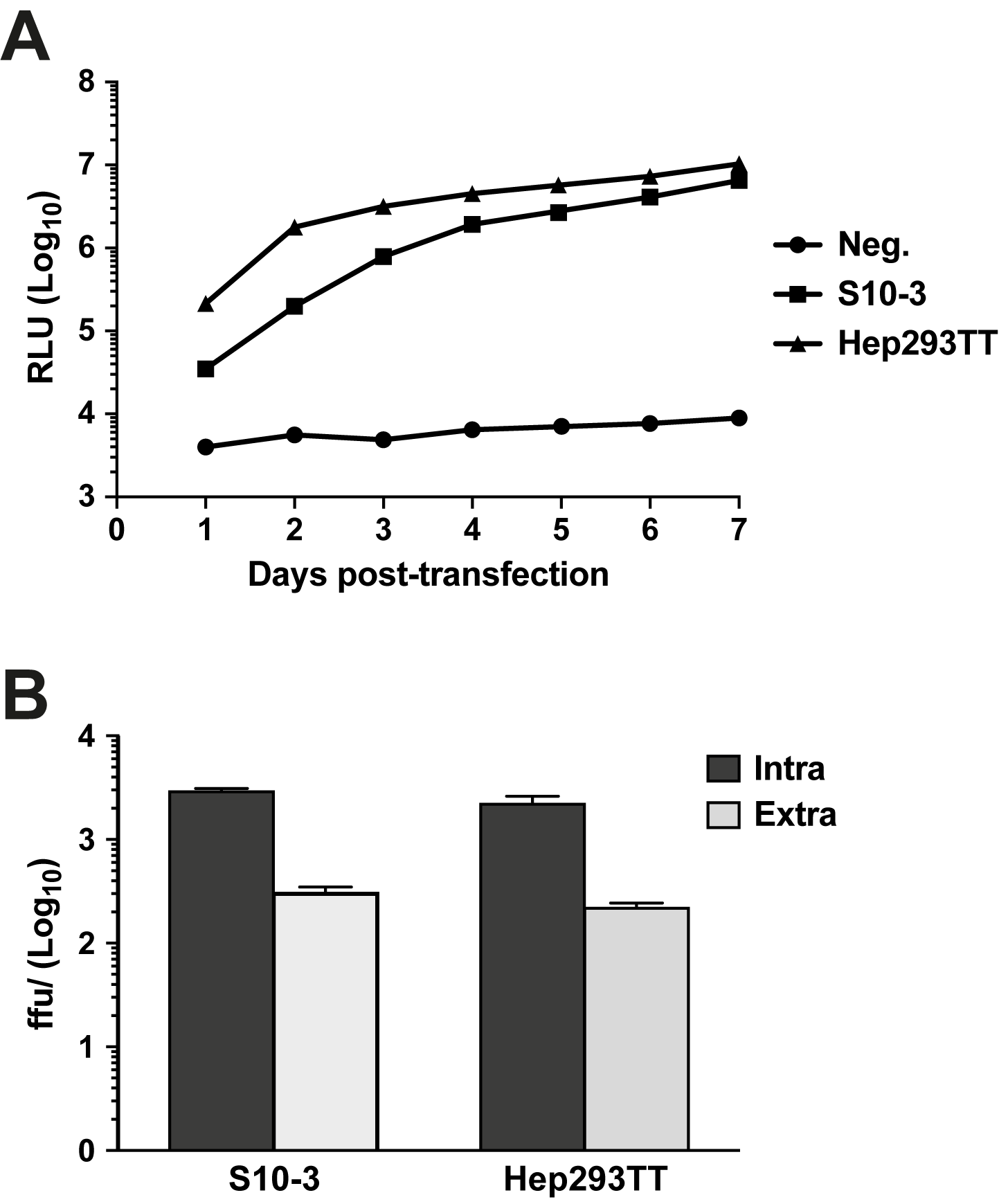

Supplement: S2 Fig — (A) Hep293TT cells are replicating HEV subgenomic replicon. S10-3 and Hep293TT were transfected with p6-luc HEV replicon and the cell culture medium was harvested every day to measure the gaussia luciferase activity. S10-3 cells transfected with the polymerase-deficient construct p6-luc-GAD served as negative control (Neg.). (B) Hep293TT cells can produce infectious HEV particle. S10-3 and Hep293TT cells were transfected with full-length p6 HEV RNA. Five days post-transfection, culture supernatants were harvested and cell lysates were prepared by freeze-and-thaw cycles followed by clarification by centrifugation at 2,000 g for 15 min Intracellular (Intra) and extracellular (Extra) infectivities were determined by foci forming assay with HepG2/C3A cells using respectively, the cell lysates and the culture supernatants as inoculum. Immunofluorescence detection of the capsid protein was performed with mAb 1E6 against HEV ORF2. ffu: focus forming unit. (TIF) [file ppat.1007471.s003.tif]

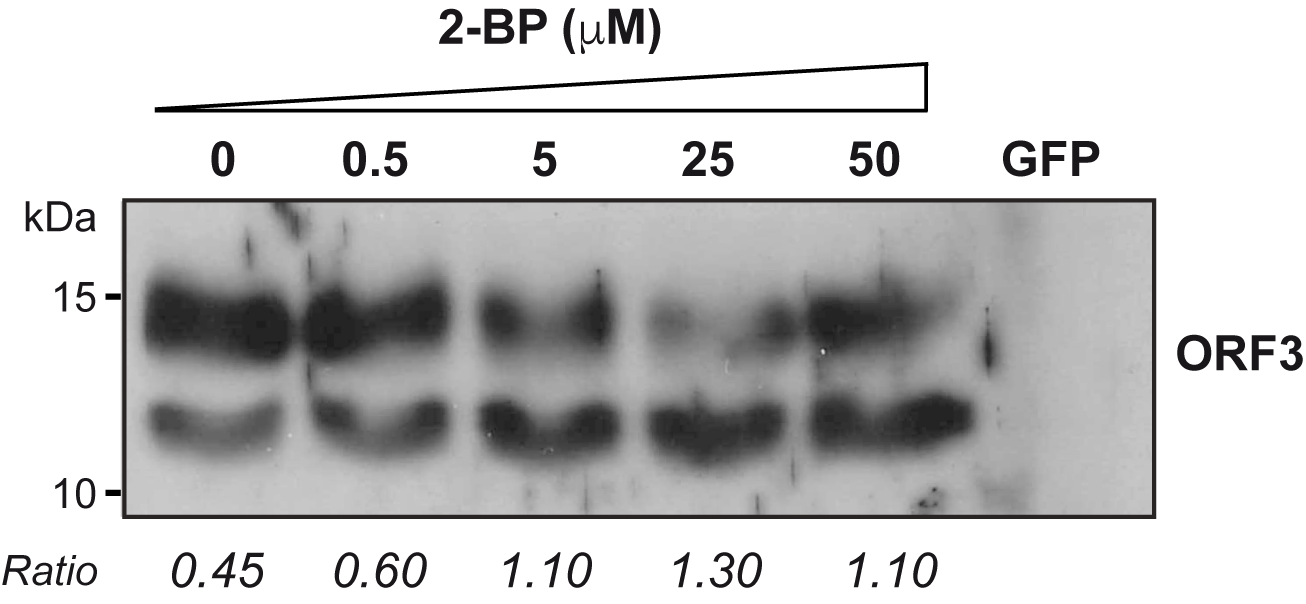

Supplement: S3 Fig — U-2 OS cells transfected with pCMVORF3 and cultured in presence of 5% FCS and with increasing concentrations of 2-BP were harvested 24 h post-transfection. Immunoblot analysis was done with pAb anti-ORF3. Corresponding lower vs upper band intensity ratio is shown below the immunoblot. (TIF) [file ppat.1007471.s004.tif]

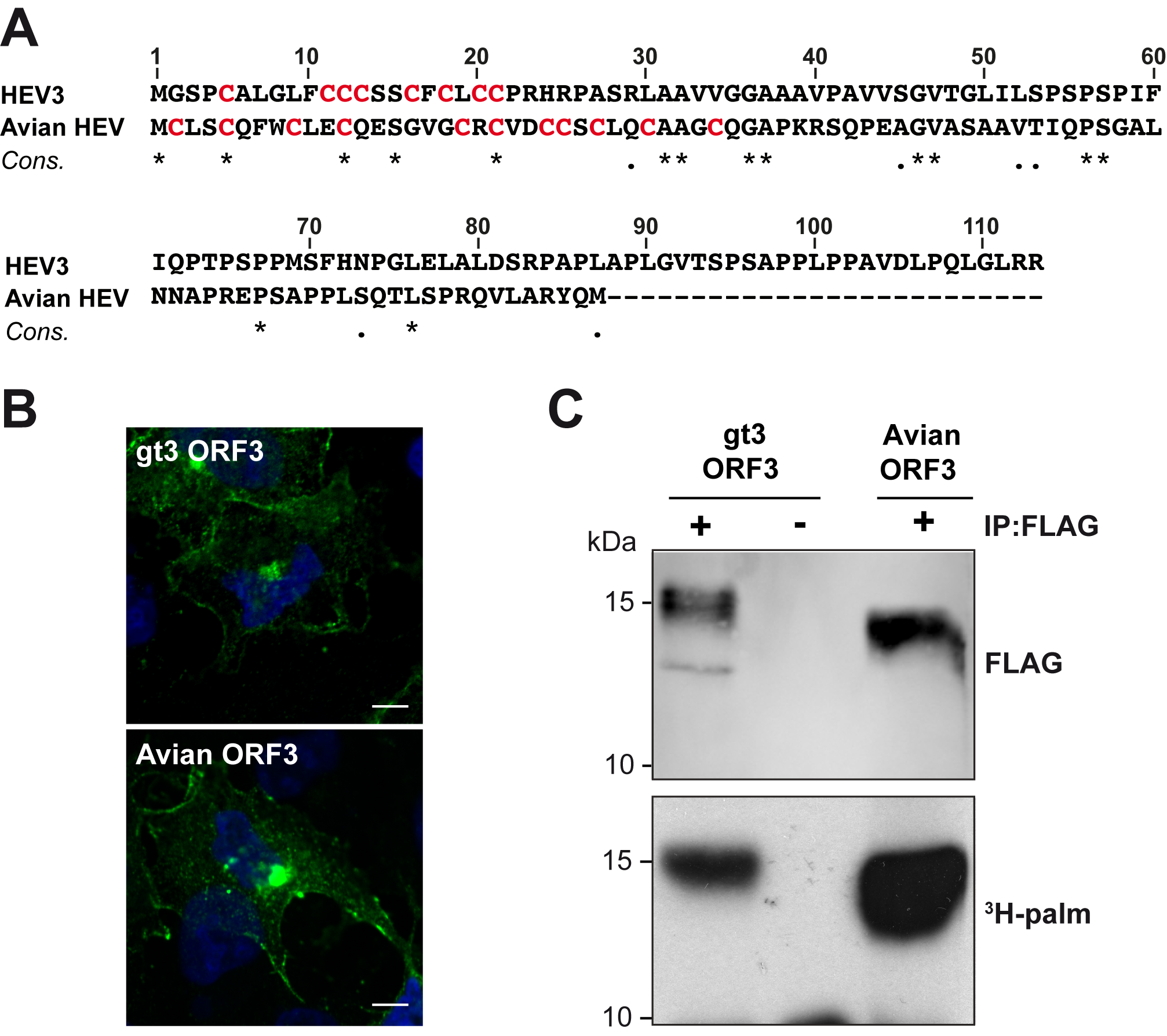

Supplement: S4 Fig — (A) Amino acid sequences of ORF3 from HEV genotype 3 (HEV3) (GenBank accession number AB740232) and avian HEV (GenBank accession number AY535004) were aligned by ClustalW. The degree of aa physicochemical conservation at each position is shown on the bottom line and can be inferred with the similarity index according to ClustalW convention (asterisk, invariant; colon, highly similar; dot, similar) [52]. (B) Subcellular localization of avian HEV ORF3. U-2 OS cells transfected with pCMVORF3-FLAG or pCMVORF3avian-FLAG were subjected to immunofluorescence using anti-FLAG M2 mAb and DAPI staining of the nucleus before confocal microscopy analysis. Scale bars indicate 10 μm. (C) S10-3 cells transfected with pCMVORF3-FLAG or pCMVORF3avian-FLAG were incubated with Dulbecco Modified Eagle Medium‎ supplemented with 3H-palmitate for 3 h. Protein lysates were prepared and subjected to imunoprecipitation with either anti-FLAG M2 mAb (+) or non-relevant mouse mAb (-). After immunoprecipitation, the elution samples were separated by 17% SDS-PAGE and subjected to either immunoblot with anti-FLAG M2 mAb followed by chemiluminescence revelation or autoradiography (40 days of exposure). (TIF) [file ppat.1007471.s005.tif]

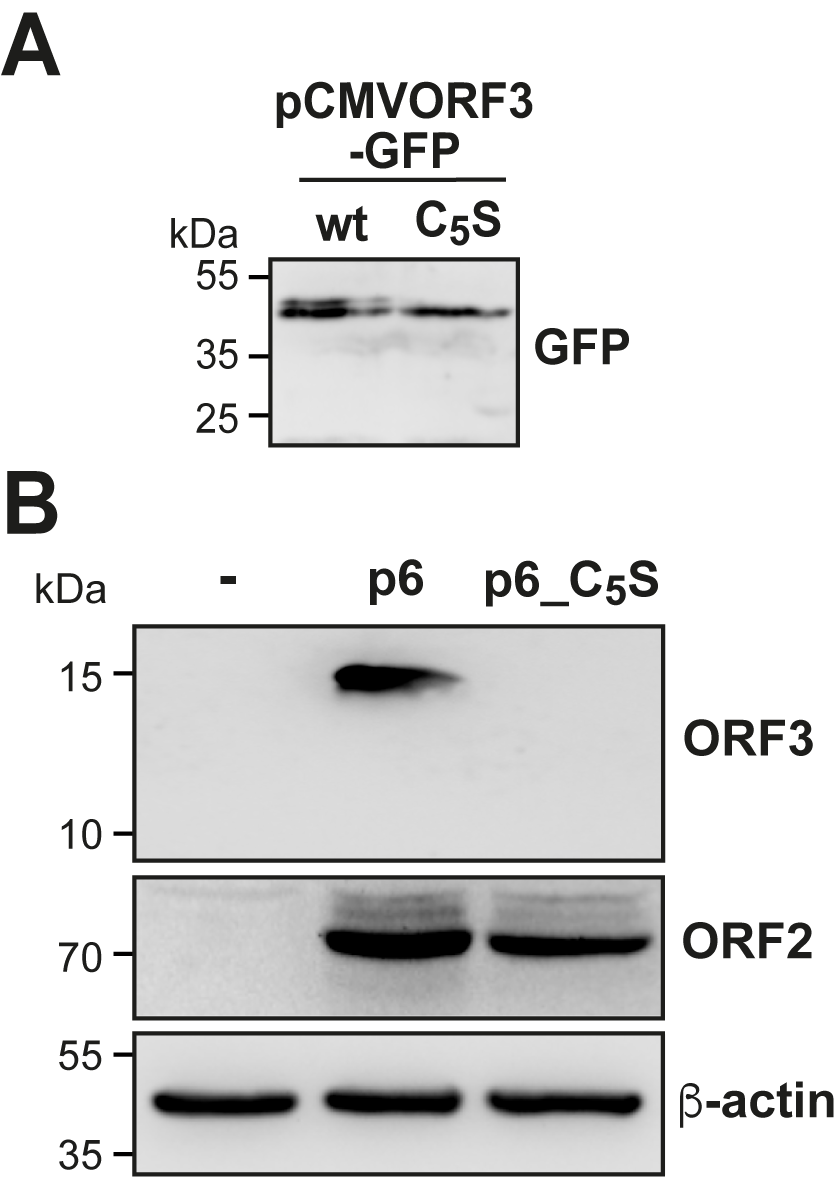

Supplement: S5 Fig — (A) S10-3 cells transfected with pCMVORF3-GFP (wt) or pCMVORF3C5S-GFP (C5S) were analyzed 48 h post-transfection by 12% SDS-PAGE followed by immunoblot with JL8 mAb against GFP. (B) Naïve Hep293TT cells (-) or Hep293TT cells replicating the full-length p6 or p6_C5S HEV RNA were lysed 6 d post-electroporation, followed by 17% SDS-PAGE and immunoblot with either anti-ORF3 pAb, anti-ORF2 mAb or anti-β-actin mAb. (TIF) [file ppat.1007471.s006.tif]
